# Improving the sensitivity of *T*_1_ contrast-enhanced MRI and sensitive diagnosing tumors with ultralow doses of MnO octahedrons

**DOI:** 10.7150/thno.59096

**Published:** 2021-05-08

**Authors:** Lijiao Yang, Lili Wang, Guoming Huang, Xuan Zhang, Lanlan Chen, Ao Li, Jinhao Gao, Zijian Zhou, Lichao Su, Huanghao Yang, Jibin Song

**Affiliations:** 1 MOE Key Laboratory for Analytical Science of Food Safety and Biology, College of Chemistry, Fuzhou University, Fuzhou 350108, China.; 2 Department of Diagnostic Radiology, Fujian Medical University Union Hospital, Fuzhou 350001, P. R. China.; 3 State Key Laboratory of Physical Chemistry of Solid Surfaces, The MOE Laboratory of Spectrochemical Analysis & Instrumentation, and Department of Chemical Biology, College of Chemistry and Chemical Engineering, Xiamen University, Xiamen 361005, China.; 4 State Key Laboratory of Molecular Vaccinology and Molecular Diagnosis & Center for Molecular Imaging and Translational Medicine, School of Public Health, Xiamen University, Xiamen 361102, China.

**Keywords:** factors affecting *T*_1_ CE-MRI, sensitive tumor imaging, zwitterionic ZDS coating, ultralow dose, MnO octahedrons

## Abstract

**Rationale:** Sensitive and accurate imaging of cancer is essential for early diagnosis and appropriate treatment. For generally employed magnetic resonance imaging (MRI) in clinic, comprehending how to enhance the contrast effect of *T*_1_ imaging is crucial for improving the sensitivity of cancer diagnosis. However, there is no study ever to reveal the clear mechanism of how to enhance the effect of *T*_1_ imaging and accurate relationships of influencing factors. Herein, this study aims to figure out key factors that affect the sensitivity of *T*_1_ contrast-enhanced MRI (CE-MRI), thereby to realize sensitive detection of tumors with low dose of CAs.

**Methods:** Manganese oxide (MnO) nanoparticles (NPs) with various sizes and shapes were prepared by thermal decomposition. Factors impacting *T*_1_ CE-MRI were investigated from geometric volume, surface area, crystal face to *r*_2_/*r*_1_ ratio. *T*_1_ CE-MR imaging of liver, hepatic and subcutaneous tumors were conducted with MnO NPs of different shapes.

**Results:** The surface area and occupancy rate of manganese ions have positive impacts on the sensitivity of *T*_1_ CE-MRI, while volume and *r*_2_/*r*_1_ ratio have negative effects. MnO octahedrons have a high *r*_1_ value of 20.07 mM^-1^s^-1^ and exhibit an excellent enhanced effect in liver *T*_1_ imaging. ZDS coating facilitates tumor accumulation and cellular uptake, hepatic and subcutaneous tumors could be detected with MnO octahedrons at an ultralow dose of 0.4 mg [Mn]/kg, about 1/10 of clinical dose.

**Conclusions:** This work is the first quantitative study of key factors affecting the sensitivity of *T*_1_ CE-MRI of MnO nanoparticles, which can serve as a guidance for rational design of high-performance positive MRI contrast agents. Moreover, these MnO octahedrons can detect hepatic and subcutaneous tumors with an ultralow dose, hold great potential for sensitive and accurate diagnosis of cancer with lower cost, less dosages and side effects in clinic.

## Introduction

The sensitive imaging of tumors is vital for early diagnosis, appropriate treatment and accurate prognosis. Among various clinical diagnostic techniques, magnetic resonance imaging (MRI) has been generally employed due to its noninvasive feature, exquisite soft tissue contrast, unlimited tissue penetration depth and high spatial resolution 1-3. Compared with *T*_2_-weighted MRI, *T*_1_ imaging has obvious advantages because positive signals can avoid confusion in recognizing lesions from normal hypointense tissues 4, 5, and the interference caused by calcification, blood pooling and metal deposition 6. Sensitive diagnosis in *T*_1_ imaging in clinic usually requires the utilization of contrast agents (CAs), which can effectively shorten the local spin-lattice relaxation time of protons, and thus raise the longitudinal relaxation rate of water molecules in their vicinity, resulting in greater contrast between different biological tissues 7.

*T*_1_ CAs primarily consist of paramagnetic metal based chelates and nanoparticles (NPs), such as gadolinium-based CAs 8, 9, manganese-based CAs 10, 11 and ultrasmall iron oxide NPs 12, 13. Among them, gadolinium-based chelates were associated with a devastating and latent fatal condition called nephrogenic systemic fibrosis (NSF) 14, the onset of NSF occurs months after the last administration in patients with poor kidney function. Meanwhile, the US Food and Drug Administration (FDA) disclosed that the use of gadolinium-based CAs would induce gadolinium deposition in the brain or bone 15, 16. On the other hand, ultrasmall iron oxide NPs as *T*_1_ CAs attract lots of attention due to their suppressed magnetization by the strong surface spin-canting effect 17, 18. There are also many excellent surface ligand modifications, such as phosphoric acid anchoring group, carboxyl anchoring group, and catechol anchoring group can can avoid the dissolution and oxidation of ultrasmall iron oxide NPs into Fe ions in biological media 19-21. However, ultrasmall iron oxide NPs are usually ultrasmall spheres and not easy for further shape engineering and investigating. By contrast, manganese-based NPs with high stability and relatively good biocompatibility are extensively employed in *T*_1_ imaging in MRI 22-26.

Though numerous nanomaterials have been broadly developed for improving the sensitivity of* in vitro* and *in vivo T*_1_ imaging, the clear mechanism of how to enhance the effect of *T*_1_ imaging is still unresolved. The Solomon, Bloembergen, and Morgan (SBM) theory elucidates the mechanism of relaxation for metal chelates 27-30. But for nanomaterials, it would be inapplicable to employ this theory because of the complexity of surface structure and the uncertainty in chemical coordination. Previous research mainly focused on particular aspects of *T*_1_ CAs, *e.g.*, size and surface structure 31-33, or based on an ideal solid model without sophisticated deep sunken structures and a complicated system with the random position of two metals in some space lattice 34. In addition, they all only qualitatively studied these influencing factors, the accurate relationship among them still needs to be further explored. Therefore, it is urgent to establish a systematic and comprehensive mechanism for understanding crucial factors that affect *T*_1_ contrast-enhanced (CE) effect for magnetic nanomaterials, thereby improves the sensitivity of *T*_1_ imaging.

**Scheme 1:**
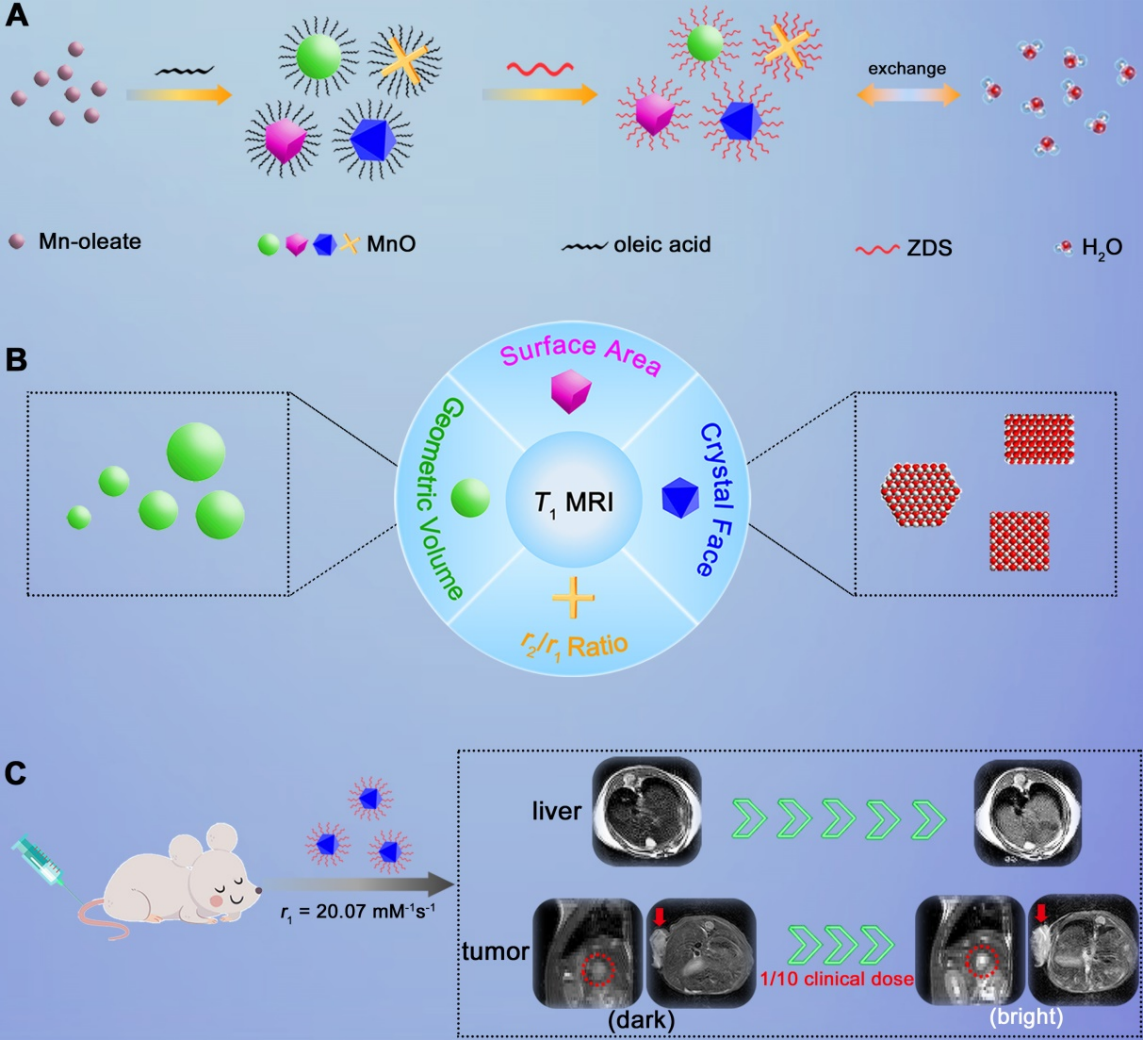
** Schematic illustration of investigating factors that affect the sensitivity of *T*_1_ imaging. (A)** Preparation of ZDS modified MnO NPs with different shapes (sphere, cube, octahedron and cross), and the proton exchange of NPs with surrounding water molecules. **(B)** Factors that impact the contrast effect of *T*_1_ imaging: geometric volume, surface area, crystal face and *r*_2_/*r*_1_ ratio.** (C)** Significant *in vivo* liver imaging enhancement after intravenously injecting MnO octahedrons with a high *r*_1_ value of 20.07 mM^-1^s^-1^, and sensitive contrast-enhanced MRI of hepatic and subcutaneous tumors with intravenous injection of MnO octahedrons at 1/10 of clinical dose.

Herein, we adopted manganese oxide (MnO), a pervasively applied agent, as research subject and investigated elements that impact *T*_1_ enhanced effect (**Scheme 1**). We prepared MnO NPs with different sizes (11-25 nm) and shapes (sphere, cube, octahedron and cross). For given nanomaterials, besides the surface modification reported, factors that affect the contrast effect of *T*_1_ imaging include geometric volume, surface area, crystal face and *r*_2_/*r*_1_ ratio. These factors can influence the intensity of paramagnetic ions on exposed surfaces and the effective chemical exchange of water protons with surface paramagnetic ions. High occupancy rate of metal ions on exposed crystal surface and large surface area would lead to high longitudinal relaxivity rate, while large geometric volume results in low *T*_1_ relaxation rate and high *r*_2_/*r*_1_ ratio has negative effect on *T*_1_ contrast effect. Moreover, it is noteworthy that MnO NPs with octahedron shape have a markedly high *r*_1_ value of 20.07 mM^-1^s^-1^ and a low *r*_2_/*r*_1_ ratio of 1.94, which endows strong contrast effects in *in vitro* and liver imaging. Meanwhile, with zwitterionic ZDS coating, these octahedrons exhibited strong signal enhancement in hepatic and subcutaneous tumor imaging at an ultralow dose of only 0.4 mg/kg, possessing great potential in sensitive and precise diagnosis in cancer.

## Methods

### Materials and characterization

Manganese chloride tetrahydrate (99%), oleic acid (tech, 90%) and 1-Octadecene (tech, 90%) were purchased from Sigma-Aldrich (USA). Sodium oleate was purchased from Sinopharm Chemical Reagent Co., Ltd. All reagents were used without further purification.

Transmission electron microscopy (TEM) and the related high-resolution TEM (HRTEM) images were performed on a FEI Tecnai G2 F20 microscope (accelerating voltage, 200 kV) and a Hitachi HT7700 Exalens microscope (accelerating voltage, 120 kV). The X-ray powder diffraction (XRD) patterns of the nanoparticles were acquired on a D/MAX-Ultima VI X-ray powder diffractometer (Rigaku Co., Japan). The X-ray absorption spectra (XPS) were conducted at an Escalab 250Xi X-ray photoelectron spectrometer (Thermo Scientific). The hysteresis loops at 300 K were recorded by the superconducting quantum interference device (SQUID). Dynamic light scattering (DLS) were measured by Zetasizer Nano ZS (Malvern Instruments Ltd., England). The measurement of relaxivity and phantom imaging at 0.5 T were all performed on an NMI20-Analyst system. *In vivo* MRI were performed on 7 T micro MRI System. The concentrations of metals were measured by inductively coupled plasma mass spectroscopy (ICP-MS) on an iCAP RQ system (Thermo Fisher).

### Synthesis of manganese oleate complexes

The manganese oleate complexes were synthesized by reacting manganese chlorides and sodium oleate following the typical method. 0.629 g (5 mmol) manganese chloride and 3.044 g (10 mmol) sodium oleate were mixed in 20 mL ethanol and 20 mL distilled water. The solution was heated to 70 °C and maintained for 4 h with stirring under N_2_ atmosphere. Then the upper layer containing manganese oleate (pink waxy) was separated. Hexane was added and then the solution was washed by water three times. After vaporizing hexane, the manganese oleate was dissolved in 1-octadecene and sealed to avoid oxidation.

### Preparation of MnO nanoparticles

We used a one-pot synthesis method to produce MnO NPs with different shapes and sizes. We strictly controlled the amount of oleic acid, sodium oleate, heated temperature and the reflux time. For spheres, 0.618 g (1 mmol) manganese oleate and 0.161 mL (0.5 mmol) oleic acid were mixed in 10 mL of 1-octadecene. The solution was first heated at 100 ºC for 20 min in vacuum to remove impurities with low boiling points in air and solvent. Then the solution was slowly heated, maintained at 200-250 °C for 30 min, and then refluxed at 320 °C in a N_2_ atmosphere before cooling to room temperature. The refluxing time was 1 h, 1.5 h, 2 h, 2.5 h and 3 h for spheres of 11 nm, 15 nm, 19 nm, 22 nm and 25 nm, respectively. For cubes, 0.618 g manganese oleate and 0.161 mL oleic acid were mixed in 15 mL of 1-octadecene. After removing impurities at 100 ºC, the solution was heated to 330 °C rapidly and maintained at this temperature for 2 h in N_2_. For octahedrons, 0.618 g manganese oleate, 0.061 mg sodium oleate and 0.161 mL oleic acid were mixed in 12 mL of 1-octadecene. After removing impurities, the solution was heated to 350 °C rapidly and refluxed for 1.5 h in N_2_. For cross, 0.618 g manganese oleate and 0.322 mL oleic acid were mixed in 15 mL of 1-octadecene with the adding of 0.152 mg sodium oleate. Then the solution was heated to 300 °C with a constant heating rate of 5 °C min^-1^ and refluxed for 4 h in N_2_. After the solution was cooled down to room temperature, all the products were separated by centrifugation, washed with ethanol for three times and dispersed in hexane for further use.

### Synthesis of zwitterionic dopamine sulfonate (ZDS)

Firstly, 6 mmol dopamine hydrochloride was dissolved in 150 mL ethanol. After slowly adding 6.5 mmol of 1,3-propanesultone and 3 mmol of ammonium hydroxide (28% in water) under N_2_ atmosphere, the resulting solution was heated at 50 °C for 18 h. Then the dopamine sulfonate was yielded by collecting the white precipitate and washing with ethanol for three times. Afterward, 1 mmol of dopamine sulfonate and 2.4 mmol of anhydrous sodium carbonate were dissolved in 150 mL dimethylformamide (DMF) with the adding of 35 mmol of iodomethane in N_2_. The resulting solution was heated to 50 °C and stirred at that temperature for 8 h to obtain a yellow oily mixture after removal of DMF in vacuum. After adding 50 mL DMF/Ethyl acetate (1:10 v/v), a pale crude solid was precipitated. Finally, a white solid (ZDS) was acquired by washing with 50 mL refluxing DMF/acetone (1:10 v/v) for three times.

### Preparation of water soluble ZDS coated nanoparticles

4 mL hexane containing about 10 mg as-prepared MnO NPs, 4 mL acetone, 2 mL deionized water and 10 mg zwitterionic dopamine sulfonate (ZDS) was mixed in a nitrogen atmosphere. The resulting solution was stirred at room temperature for 4 h to undergo a ligand exchange process. Then the ZDS coated NPs were collected by centrifugation, dispersed in deionized water and stored at 4 °C for further use.

### Cytotoxicity evaluation

3-(4,5-dimethylthiazol-2-y1)-2,5-diphenyltetrazolium bromide (MTT) assays was used to evaluate the cytotoxicity of ZDS coated MnO NPs of different shapes and sizes with SMMC-7721 cells. Cells were first seeded into a 96-well plate in RPMI 1640/DMEM at a density of 1 × 10^4^ cells/well and incubated at 37 °C under 5% CO_2_ overnight. Cells were incubated with ZDS coated MnO NPs for 24 h at different [Mn] concentrations. (0.469, 0.938, 1.875, 3.75, 7.5, 15, 30, 60, and 120 μg/mL). After adding each well with 100 μL fresh media containing 0.5 μg/mL MTT, cells were further incubated at 37 °C for 4 h. The OD_492_ value (Abs.) of each well was immediately obtained from MultiSkan FC microplate reader and accordingly the cell viability was calculated.

### *T*_1_ and *T*_2_ relaxivities measurements and *T*_1_- and *T*_2_-weighted phantom images

The *T*_1_/*T*_2_ relaxation times and *T*_1_ phantom images were conducted on a 0.5 T NMI20-Analyst NMR system. *T*_1_ phantom images were acquired with MnO NPs with different manganese concentrations of 0.4, 0.2, 0.1, 0.05, 0.025 mM and 0 mM (water). The *r*_1_ and *r*_2_ values were calculated from the slopes of the best fitting lines of 1/T versus concentration.* T*_1_- and *T*_2_-weighted phantom images were obtained with a 2D multislice spin-echo (MSE) sequence: TR/TE = 200/2 ms (*T*_1_), TR/TE = 2000/40 ms (*T*_2_), 512 × 512 matrices.

### *In vivo* liver MR imaging

*In vivo T*_1_ imaging of liver was carried out with male BALB/c mice (18-22 g, purchased from Shanghai SLAC Laboratory Animal Co., Ltd) as the model on a 7 T MRI system. All animal experiments were performed in accordance to the protocol approved by the Institutional Animal Care and Use Committee of Fuzhou University and the guide for the care and use of laboratory animals (Ministry of Science and Technology of China, 2006). The images of the liver in the transverse plane at 0 h (Pre-injection), 0.5 h, 1 h, 2 h, and 4 h were attained after intravenous injection of MnO NPs with a dose of 2.0 mg [Mn]/kg (*n* = 3/group). Parameters of fSEMS sequence: TR/TE = 500/12 ms, FOV = 40 × 40 mm, thickness = 1 mm, average = 4. Signal-to-noise ratio (SNR) was calculated by the equation: SNR_liver_ = SI_liver_/SD_noise_, where SI means signal intensity and SD represents standard deviation. The SNR change (∆SNR) was defined as ∆SNR = |SNR_post_ - SNR_pre_|/SNR_pre_.

### *In vivo* hepatic tumors MRI

All animal experiments were conducted according to the protocol approved by Institutional Animal Care and Use Committee of Fuzhou University. The mice were inoculated with an injection of 3-5 × 10^5^ H22 cells in the liver. Two weeks later, the MR images of hepatic tumors in the sagittal plane were obtained at 0, 1, 2 and 4 h after intravenous injection of MnO octahedrons with a dose of 1.0 mg [Mn]/kg and 0.4 mg [Mn]/kg, and MnO cross at a dose of 2.0 mg [Mn]/kg, and Mn-DPDP with an injection dose of 4.0 mg [Mn]/kg, respectively (*n* = 3/group). The parameters of imaging were TR/TE = 400/10 ms, thickness = 1.5 mm, and slice = 8. The contrast-to-noise ratio (CNR) changes of tumor is defined as CNR = (SNR_tumor_ - SNR_liver_) /SNR_tumor_.

### Subcutaneous tumors MR imaging

The subcutaneous tumor model of BALB/c mouse was established by injection of 5 × 10^6^ H22 cells to the subcutaneous tissue. MR images of tumor at the transverse plane at 0 h and 2 h were acquired after intravenously injecting MnO Octahedrons with a dose of 0.4 mg [Mn]/kg, MnO cross with a dose of 2.0 mg [Mn]/kg, and Mn-DPDP at a dose of 4.0 mg [Mn]/kg (*n* = 3/group). Parameters of scanning sequence: TR/TE = 500/12 ms, FOV = 40 × 40 mm, thickness = 1 mm, 256 × 256 matrices. SNR was calculated by the equation: SNR_tumor_ = SI_tumor_/SD_noise_. The contrast-to-noise ratio (CNR) changes of tumor is defined as CNR = (SNR_tumor_ - SNR_liver_) /SNR_tumor_. All the experiments were carried out in accordance with the protocol approved by Institutional Animal Care and Use Committee of Fuzhou University.

### Statistical analysis

The statistical difference was evaluated with Student's t test. The sizes of nanoparticles were acquired by measuring at least two hundred particles per sample via Image J. All data were presented as mean ± standard deviation.

## Results

### Synthesis and characterization of MnO NPs with different shapes

**Figure 1:**
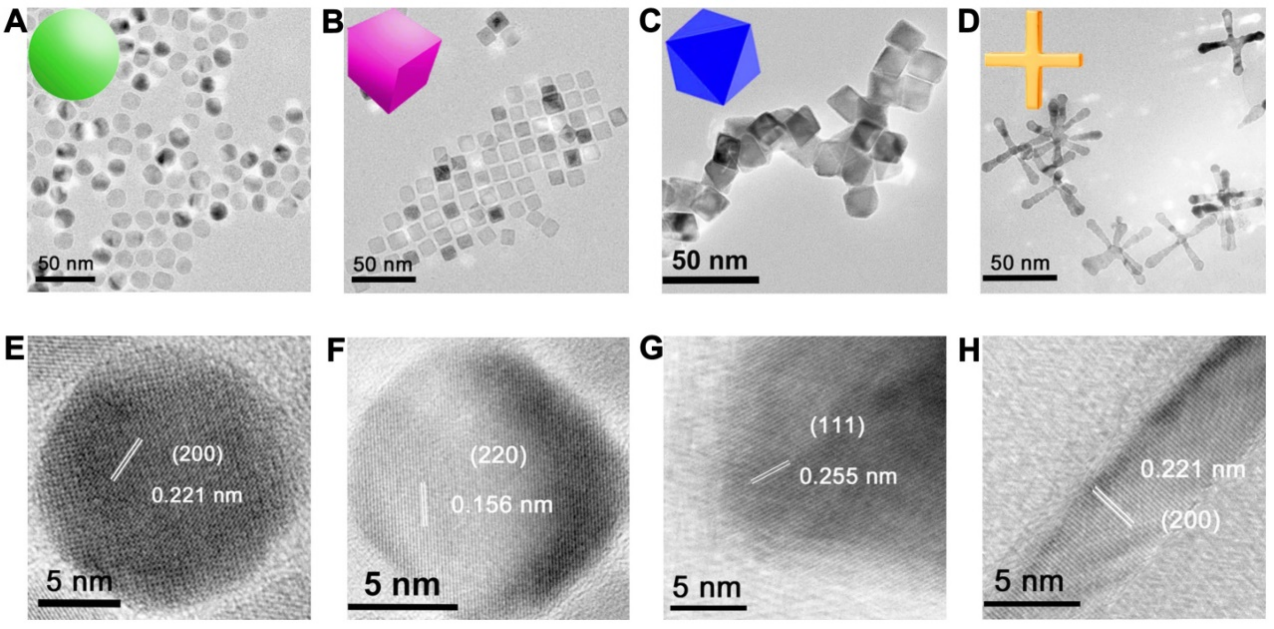
** TEM and HRTEM images of monodispersed MnO NPs of different shapes with a similar geometrical volume.** TEM images of **(A)** spheres (diameter of 15 nm), **(B)** cubes (side length of 12 nm), **(C)** octahedra (side length of 16 nm) and **(D)** cross (length of 50 nm, diameter of 5 nm). The numbers were averages calculated from two hundred nanoparticles for all samples via Image J analysis. **(E-H)** The corresponding HRTEM images with clear lattice distances of the above images.

We prepared MnO NPs using a modified one-pot synthesis method 35 by thermal decomposition of manganese oleate as precursor and oleic acid as surfactant in 1-octadecene solvent. MnO NPs with different sizes were fabricated by varying reflux time in procedural heating, MnO NPs with different shapes were prepared by controlling the reflux time and the amount of sodium oleate in different procedural heating (details see Method and **Table S1**). Transmission electron microscopy (TEM) images (**Figure 1A-D** and **Figure S1**) showed that all NPs of four shapes were uniform with high yields (>90%). They were spheres (actually polyhedrons, with a diameter of 15 nm), cubes (with a side length of 12 nm), octahedrons (with a side length of 16 nm) and cross (with a length of 50 nm and a diameter at bottom surface of 5 nm) (**Figure S2**). The high-resolution TEM (HRTEM) images (**Figure 1E-H**) showed clear lattice distances of 0.221 nm, 0.156 nm and 0.255 nm, which could be assigned to the (200), (220) and (111) facets of MnO, respectively. Spheres and cross are mainly exposed by plenty of small (200) facets, which are formed to minimize the total surface energy at relatively low temperature according to the previous report 36. While at high temperature, octahedrons with large (111) face with high surface-energy ratio are generated, as high temperature provides sufficient energy for NPs to grow along the {100} surface 37, 38. Cubes displayed an interplanar distance of 0.156 nm along the [100] zone axis, which could be ascribed to the (220) plane 39.

**Figure 2:**
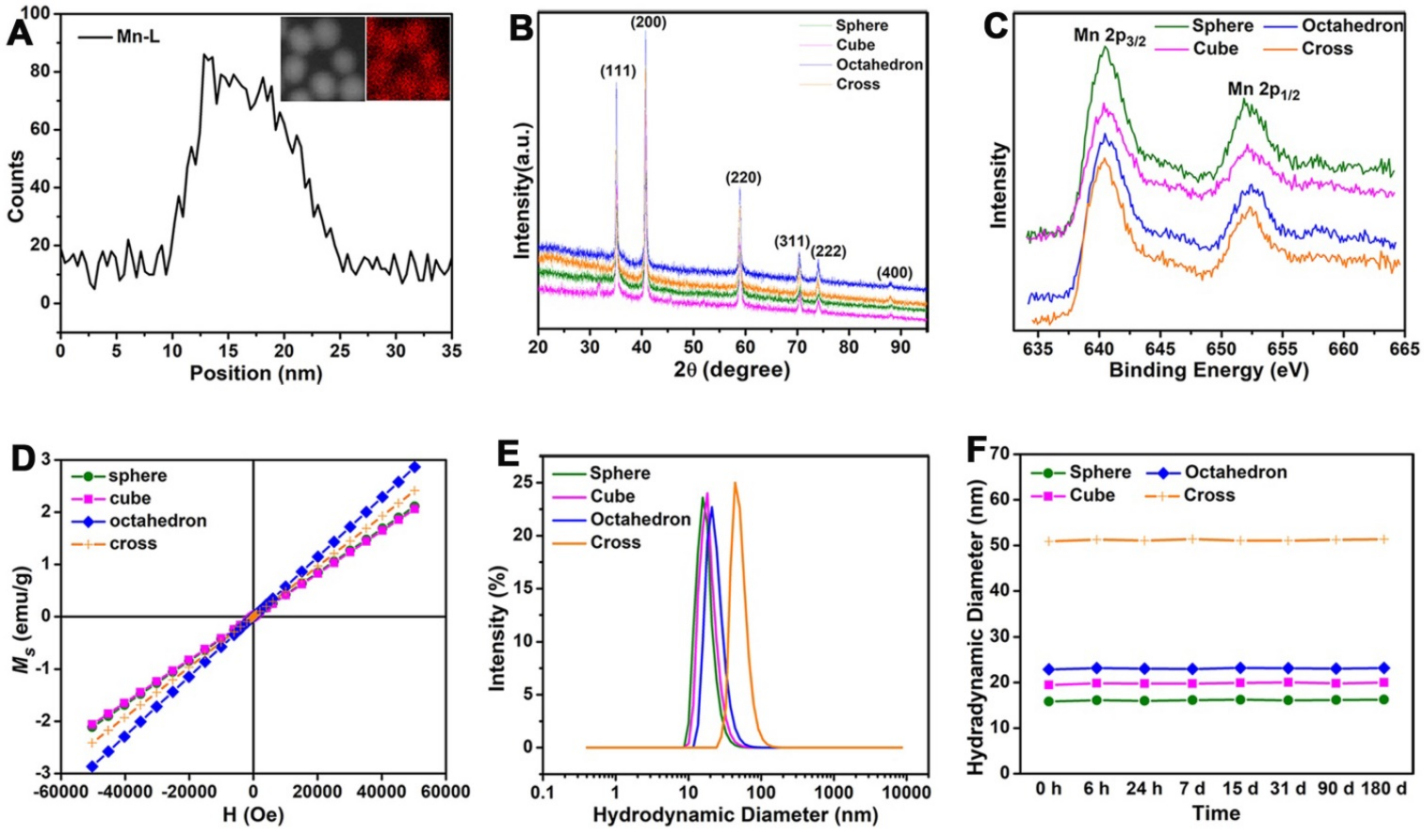
**Structure, Magnetism and Stability of MnO nanoparticles with different shapes. (A)** Energy-dispersive X-ray (EDX) elemental line scanning analysis (inset, STEM-HAADF image and EDX mapping image).** (B)** X-ray powder diffraction (XRD) patterns of MnO nanoparticles with four different shapes. **(C)** The X-ray photoelectron spectroscopy (XPS) spectra analysis of MnO nanoparticles with different morphologies. **(D)** Field-dependent magnetization (*M-H*) curves (from -60000 to 60000 Oe) of MnO NPs with four different shapes at 300 K.** (E)** Hydrodynamic diameter analysis by dynamic light scattering (DLS) measurements after surface modification with ZDS. **(F)** The long-term (from 6 hours to 180 days) hydrodynamic diameters in PBS.

The energy-dispersive X-ray (EDX) line scanning analysis and mapping image of the representative sphere (**Figure 2A**) confirmed that manganese ions were evenly distributed in MnO NPs. The diffraction peaks of X-ray powder diffraction (XRD) demonstrated the typical cubic structures (JCPDS no. 01-075-0625) of all four MnO samples (**Figure 2B**). The peaks at 35.10, 40.76, 59.01, 70.55, 74.19 and 88.29 are assigned to (111), (200), (220), (311), (222) and (400) planes of cubic MnO, respectively. Consistent with the XRD analysis, X-ray photoelectron spectroscopy (XPS) spectra (**Figure 2C**) showed clear peaks of Mn 2p_3/2_ at 640.33 eV, 640.36 eV, 640.34 eV and 640.43 eV for spheres, cubes, octahedrons and cross, respectively, indicating the existence of pure Mn(II) without Mn(III) in these MnO NPs 40. The field-independent magnetization (*M-H*) curves (**Figure 2D**) affirmed that magnetic moments of four nanostructures exhibited linear trends with the applied magnetic fields at room temperature (300 K), indicating that all MnO performed typical paramagnetic behaviors owing to the existence of uncompensated spins on the surface of particle 41, 42.

The majority of small molecule ligands only show certain positive or negative surface charge, which is unfavorable to the *in vivo* pharmacokinetics and *T*_1_ imaging of NPs. NPs with negatively charged surface face a limitation in the efficient theranostic response and appear to negatively affect the internalization of NPs, while positively charged NPs are easily cleared from blood circulation 43-45. Therefore, we chose zwitterionic dopamine sulfonate (ZDS) with neutral charge as surface coating ligand for phase transfer. TEM images (**Figure S3**) and size distributions (**Figure S4**) of ZDS-coated MnO NPs with different shapes showed there is no change in their shapes and diameters after ZDS coating. Zeta potential analyses indicate that MnO nanoparticles with diverse shapes possess neutral surface charges in water (**Figure S5**). Dynamic light scattering (DLS) analysis (**Figure 2E**) confirmed that all MnO NPs after ZDS coating had narrow size distributions. The hydrated diameters (**Figure S6**) were 16.25 ± 1.92 nm, 19.98 ± 3.01 nm, 23.17 ± 2.38 nm and 51.34 ± 3.94 nm for spheres, cubes, octahedrons and cross, respectively. The polydispersity coefficient (PDI) (**Table S2**) corroborated that MnO NPs were stable in PBS solutions for more than six months. Moreover, the diameters of the particles in PBS (**Figure 2F**) barely changed for a long time, which attests the good stabilities of these ZDS coated MnO NPs.

### Investigation on factors impacting *T*_1_ MR imaging

**Figure 3:**
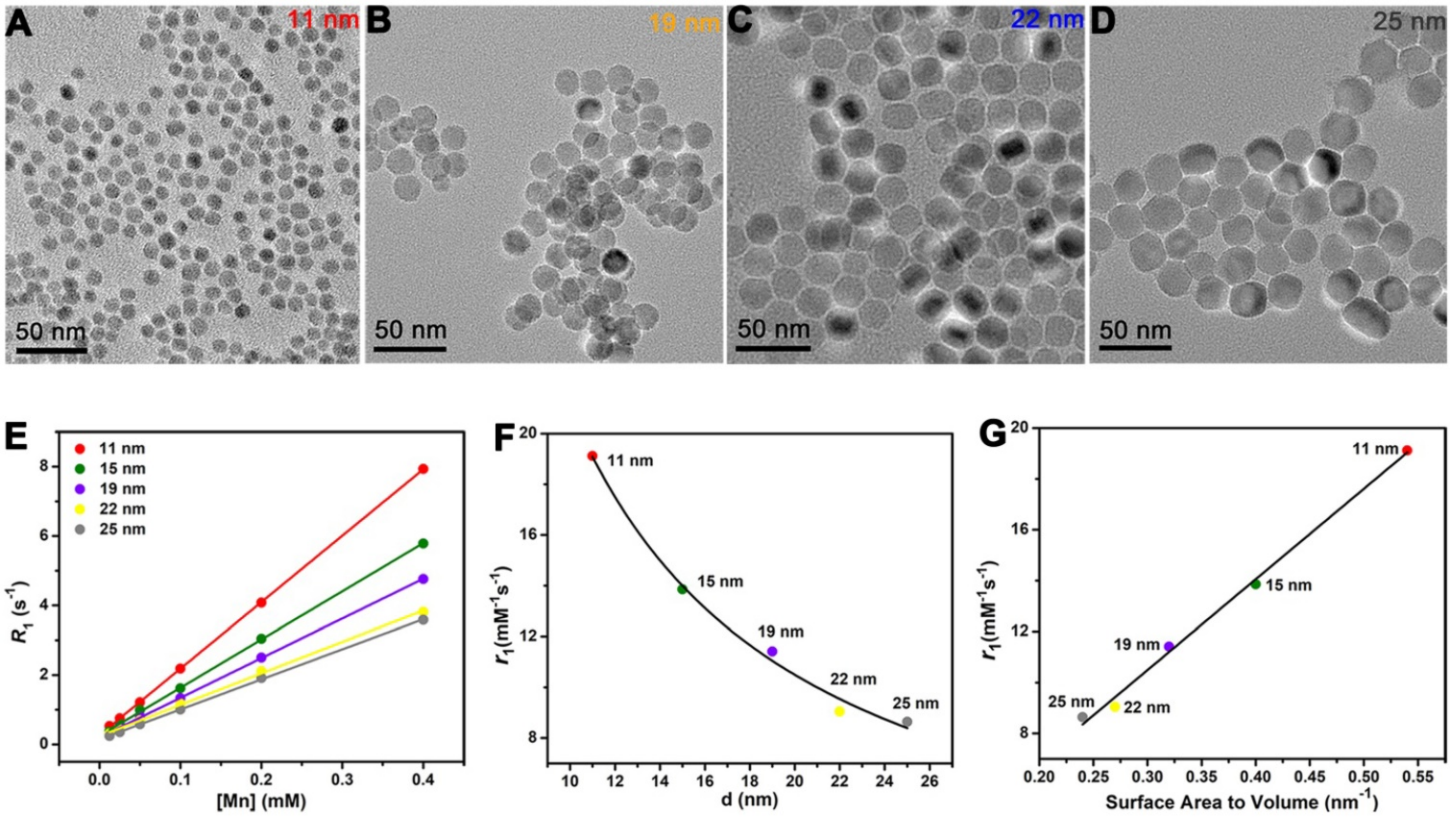
**The influences of diameter and surface area to volume ratio in *T*_1_ relaxivity.** TEM images of monodispersed MnO spheres with different sizes: **(A)** 11 nm, **(B)** 19 nm, **(C)** 22 nm and **(D)** 25 nm. Scale bar, 50 nm. The diameters were obtained by measuring of at least two hundred particles per sample *via* Image J. **(E)** The analysis of longitudinal relaxation rate *R*_1_ (1/*T*_1_) of MnO spheres with 11 nm, 15 nm, 19 nm, 22 nm and 25 nm. *T*_1_ relaxivities were calculated from the slopes of the best-fit linear lines for experimental data. **(F)** The relationship of *r*_1_ value and diameter. The solid line is the fitting curve.** (G)** The linear relationship of *r*_1_ value and surface area to volume ratio.

Size-dependent *T*_1_ relaxivity has been reported by previous studies 46, but the underlying mechanism behind this appearance is unclear. To avoid the shape effect, it needs NPs have the same surface structure and surface modification. Hence, besides the 15 nm sphere, we also prepared MnO spheres with other different sizes (**Figure 3A-D**). Their diameters were 11 nm, 19 nm, 22 nm and 25 nm (**Figure S7**). We then tested their *T*_1_ relaxivities on a 0.5 T MR scanner (**Figure 3E**). The *r*_1_ values of 11 nm, 15 nm, 19 nm, 22 nm and 25 nm were 19.12 ± 0.33, 13.86 ± 0.35, 11.41 ± 0.38, 9.05 ± 0.25 and 8.64 ± 0.34 mM^-1^s^-1^, respectively (**Figure S8**). Notably, the *T*_1_ relaxivity had a decreasing trend with the increase of the size. Thereupon, we analyzed their relationships between *r*_1_ values and diameters of these spheres. The *T*_1_ relaxivity is inversely proportional to the diameter, and the nonlinear correlation coefficient is 0.991 (**Figure 3F**), which indicates an outstanding nonlinear relationship. Coincidentally, for spheres, the diameter has a reciprocal relationship of the surface area to volume ratio. We then further investigated their relationships between *r*_1_ values and surface area to volume ratios (**Table S3**). Consists with the trends of diameters, their *T*_1_ relaxivities showed upward tendencies with their surface area to volume ratios (**Figure 3G**), and the palmary linear correlation coefficient is 0.994. This phenomenon could be attributed to the fact that the surface to volume ratio can reflect the relative intensity of paramagnetic ions on exposed surfaces. In principle, the *T*_1_ relaxation increasement is primarily related to the inner sphere regime that protons directly acquire effective chemical exchange with surface paramagnetic ions 47. Thus, more paramagnetic ions are exposed on the surface with a higher surface to volume ratio, which leads to a higher *T*_1_ relaxivity enhancement.

**Figure 4:**
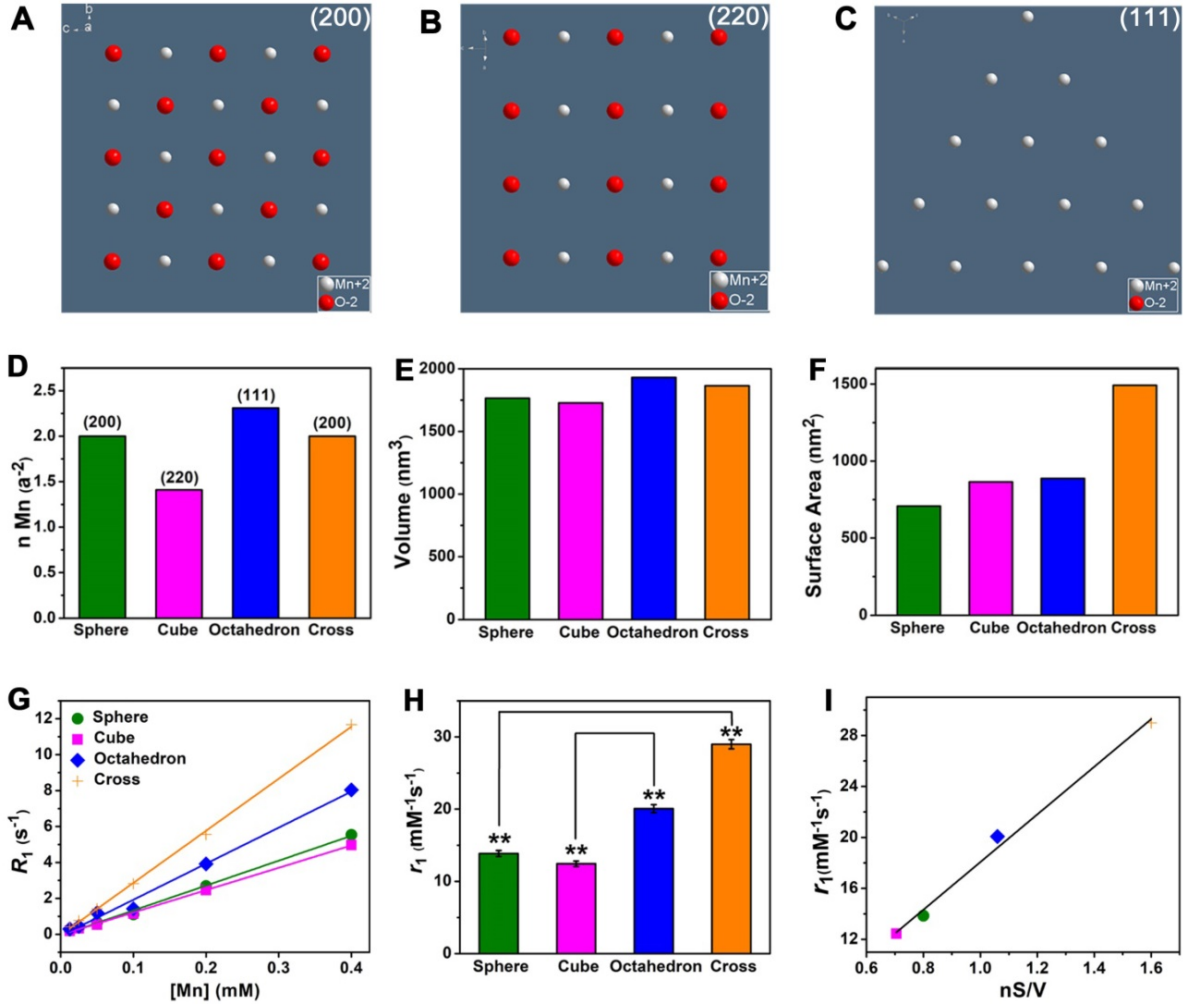
** The impacts of crystal face, surface area and geometric volume in *T*_1_ relaxivity. (A)**, **(B)** and **(C)** The exposed faces of (200), (220), and (111) of MnO NPs, they have different intensities of manganese ions on the surface. **(D)** The different occupancy rates of manganese (n Mn) on the surface, **(E)** similar geometric volumes, and **(F)** different surface areas of MnO nanoparticles with different shapes. The analysis of **(G)** longitudinal relaxation rate, *R*_1_ (1/*T*_1_) and **(H)** *T*_1_ relaxivities, the *r*_1_ values were obtained from the slopes of the linear lines. **(I)** The linear relationship of *r*_1_ value and nS/V (occupancy rate of manganese multiply by surface area and divided by volume).

For MnO NPs, the investigation of clear relationship between crystal surface and *T*_1_ relaxivity remains a great challenge in recent years, which probably due to strong metal-oxygen covalent binding and diverse crystal packing structures of NPs 48, 49. In our work, MnO spheres, cubes, octahedrons and cross have different exposed crystal faces on the surface. Because of the various arrangements of atoms, the crystal face impacts the occupancy rate of effective metal ions on the surface. The (200), (220) and (111) crystal faces of MnO exhibit different occupancy rates of metal ions (**Figure 4A-C**). The number of ions is 2.00 Mn^2+^ and 2.00 O^2-^ on the (200) face, 1.41 Mn^2+^ and 1.41 O^2-^ on the (220) face, 2.31 Mn^2+^ on the (111) face, per a^2^ (a is the side length of the unit cell) (**Figure S9** and **Table S4**). Considering O^2-^ ion has no contribution to *r*_1_ value, the order for occupancy rate of effective metal on each face is (111) > (200) > (220). And the occupancy rates of effective manganese ions (n) on exposed surfaces of spheres, cubes, octahedrons, cross (**Figure 4D**) are 2.00, 1.41, 2.31 and 2.00 per a^2^, respectively. It is noteworthy that four MnO NPs of different shapes have a similar geometrical volume (**Figure 4E**), which are 1767, 1728, 1931 and 1865 nm^3^ for spheres, cubes, octahedrons and cross (**Table S5**), respectively. However, their surface areas (**Figure 4F**) are calculated to be 707, 864, 887 and 1492 nm^2^ for spheres, cubes, octahedrons and cross, respectively (**Table S6**). We then measured their *T*_1_ relaxation rates of these four samples with different shapes at 0.5 T (**Figure 4G**). The *r*_1_ values of spheres, cubes, octahedrons and cross were 13.86 ± 0.41, 12.44 ± 0.38, 20.07 ± 0.55 and 28.99 ± 0.64 mM^-1^s^-1^, respectively (**Figure 4H**).

Since the ligand of surface modification is the same and the geometric volume is similar for these four MnO samples, their difference of *r*_1_ values could be ascribed to their different crystal structures or surface areas. For spheres and cross, they have the same (200) exposed crystal face, but they have entirely different *r*_1_ values. The *r*_1_ value of cross (28.99 mM^-1^s^-1^) is much higher than that of spheres (13.86 mM^-1^s^-1^), probably because of their different surface areas. The cross has a larger surface area (1492 nm^2^) than sphere (707 nm^2^) and shows a high *r*_1_ value, which indicates *r*_1_ value has a positive correlation with surface area. Additionally, we noticed that compared with spheres, the increase in* r*_1_ value (2.09 times) and the augment in surface area (2.11 times) of cross are almost the same. This result further suggests that *T*_1_ relaxivity has a positive proportional relationship with surface area. For cubes and octahedrons, they have similar surface areas (864 and 887 nm^2^) but distinct exposed crystal faces of (200) and (111). Cubes have an *r*_1_ value of 12.44 mM^-1^s^-1^, while octahedrons have a relatively high *r*_1_ value of 20.07 mM^-1^s^-1^. As previously mentioned, the occupancy rate of effective metal on (111) face (2.31 Mn^2+^ per a^2^) is higher than (220) face (1.41 Mn^2+^ per a^2^), which implies that the *r*_1_ value has a positive relationship with the occupancy rate of manganese ions. Similarly, we discovered that the addition in* r*_1_ value (1.61 times) and the augment in occupancy rate of manganese ions (1.64 times) of cross are nearly identical. Thus, we concluded that *T*_1_ relaxivity in direct proportion to occupancy rate of metal on the surface.

To verify our conclusions, we analyzed the relationships of occupancy rate of metal (n), surface area (S), geometric volume (V) and *T*_1_ relaxivity (*r*_1_) (**Table S7**). It is noted that *r*_1_ value is closely related to nS/V for these shapes, which certifies a good positive linear relationship with a coefficient of 0.990 (**Figure 4I**). In other words, *r*_1_ value is positively related to surface area and occupancy rate of effect metal ions but negatively related to geometric volume. A large surface area increases the total number of effective metal centers on the exposed surface. As a result, compared with sphere, cross with a larger surface area can provide more effective metal ions than sphere for chemical exchange with water protons, which accelerates the *T*_1_ relaxation process and thus shows a high *r*_1_ value. Analogously, compared with cubes, octahedrons exposed metal-rich crystal faces. The occupancy rate of manganese ions on the surface of octahedrons is much higher than that of cubes because crystal surface directly determines the amount of metal on the exposed surface of NPs 50. It is well known that* T*_1_ CAs shorten the proton longitudinal relaxation time by accelerating the chemical exchange between effective metal ions on the surface of NPs and water molecules in the surrounding. Hence, octahedrons have high density of accessible metal ions and more coordination centers, which results in a fast exchange with water molecules and improve the *T*_1_ relaxivity. Above all, these results demonstrate that *T*_1_ relaxivity is closely affected by crystal face, surface area, geometric volume.

**Figure 5:**
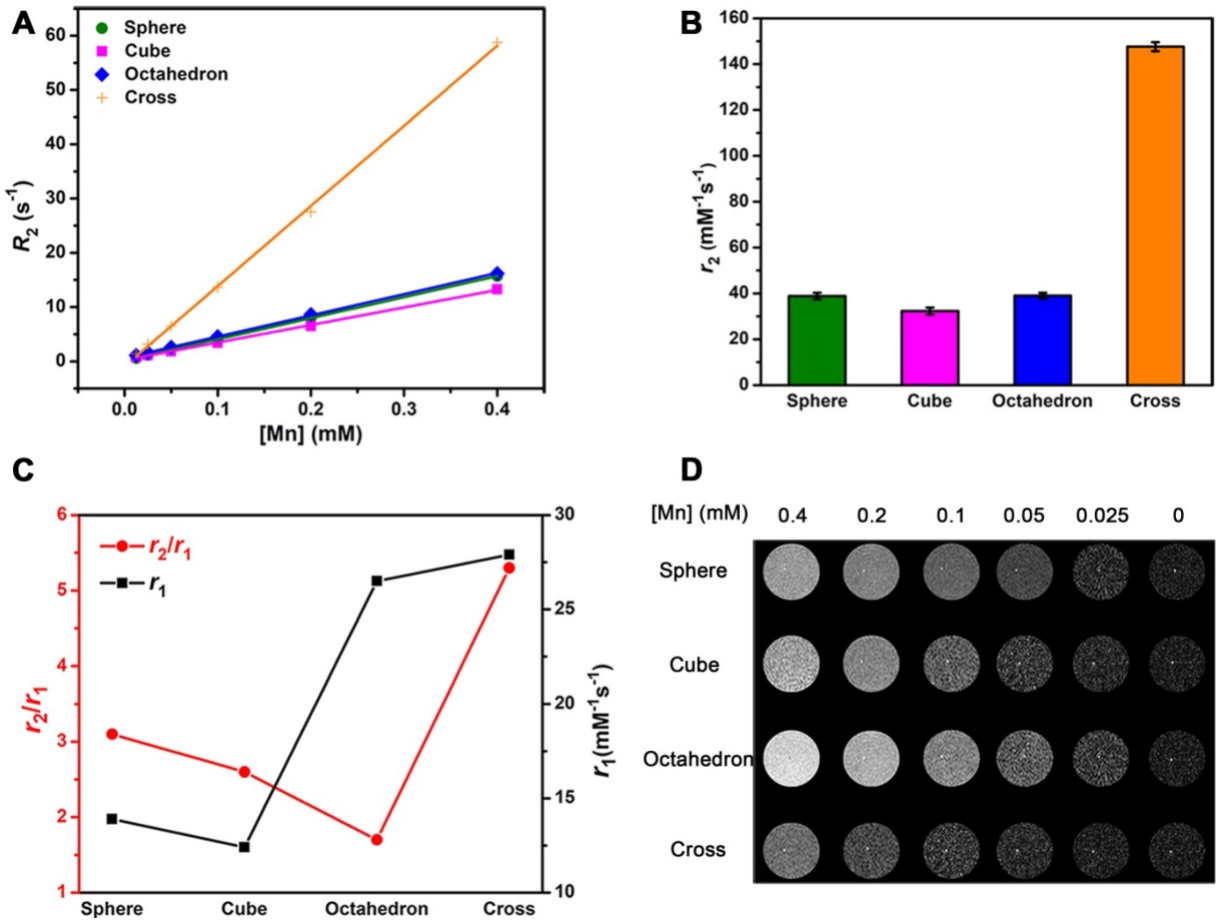
** The effect of *r*_2_/*r*_1_ ratio on *T*_1_ CE-MR imaging.** *T*_2_ relaxivity measurements of MnO nanoparticles of different shapes at 0.5 T: **(A)** Analysis of transverse relaxation rate *R*_2_ (1/*T*_2_) and** (B)** *T*_2_ relaxivities. The *r*_2_ values are acquired from the slopes of the best-fit lines. **(C)** The *r*_2_ to *r*_1_ ratios and *T*_1_ relaxivities for MnO nanoparticles with different shapes. **(D)** *T*_1_-weighted phantom images of MnO nanoparticles with four shapes at different concentrations.

Theoretically, CAs are able to accelerate both *T*_1_ and *T*_2_ relaxation process of nearby water molecules and enhance *T*_1_ and *T*_2_ signals under an external magnetic field. Thus, the *r*_2_/*r*_1_ ratio is also an important factor to estimate the effect of *T*_1_ imaging. We performed *T*_2_ relaxivity tests of these four MnO NPs with different shapes at 0.5 T (**Figure 5A**). Their *r*_2_ values were 38.81 ± 1.41, 32.28 ± 1.55, 39.02 ± 1.28 and 147.6 ± 1.97 mM^-1^s^-1^ for spheres, cubes, octahedrons and cross, respectively (**Figure 5B**). The ranking of *r*_2_ values is consistent with the trend of size of NPs, that is, a higher *r*_2_ value is obtained with a larger size, which is consistent with previous reports 51, 52. We then calculated *r*_2_/*r*_1_ ratios of these samples, they are 2.80, 2.59, 1.94 and 5.09 for spheres, cubes, octahedrons and cross, respectively (**Table S8**).

To get an intuitive sense, we studied their enhanced abilities for *T*_1_ imaging by showing their* T*_2_ relaxivities and *r*_2_/*r*_1_ ratios in one graph (**Figure 5C**). Spheres and cubes have moderate* r*_1_ values (13.86 and 12.44 mM^-1^s^-1^) and moderate *r*_2_/*r*_1_ ratios (2.80 and 2.59). Cross have the highest *r*_1_ value of 28.99 mM^-1^s^-1^ but a large *r*_2_/*r*_1_ ratio of 5.09. A high *r*_2_/*r*_1_ ratio is unfavorable for the effect of *T*_1_ imaging as a strong *T*_2_ enhanced effect would result in *T*_2_-dominated contrast. Therefore, though cross has the highest *r*_1_ value, their high *r*_2_/*r*_1_ ratio determines that they are not suitable for *T*_1_ imaging. In other words, the specific cross shape is favorable for *T*_2_ imaging. Particularly, octahedrons have a relatively high *r*_1_ value of 20.07 mM^-1^s^-1^ and a low *r*_2_/*r*_1_ ratio of 1.94, which suggests they could be a prominent agent for enhanced *T*_1_ imaging.

The results of *T*_1_-weighted phantom imaging (**Figure 5D**) further confirmed our above analysis. There was an increase of signal intensity with the augment of [Mn] concentration for all samples. Spheres and cubes showed clear *T*_1_ enhancement as the concentration increased. Nevertheless, the enhanced effect of cross was awfully weak because of its large *r*_2_/*r*_1_ ratio. Obviously, octahedrons exhibited the strongest positive enhanced effect of these NPs, which is ascribed to their high *T*_1_ relaxivity and low *r*_2_/*r*_1_ ratio. The octahedrons sample showed a palpable light signal compared with the signal of water even at the low concentration of 0.05 mM^-1^, which indicates that octahedrons have the robust ability for sensitive imaging and precise diagnosis.

### *T*_1_ CE-MR imaging of liver

**Figure 6:**
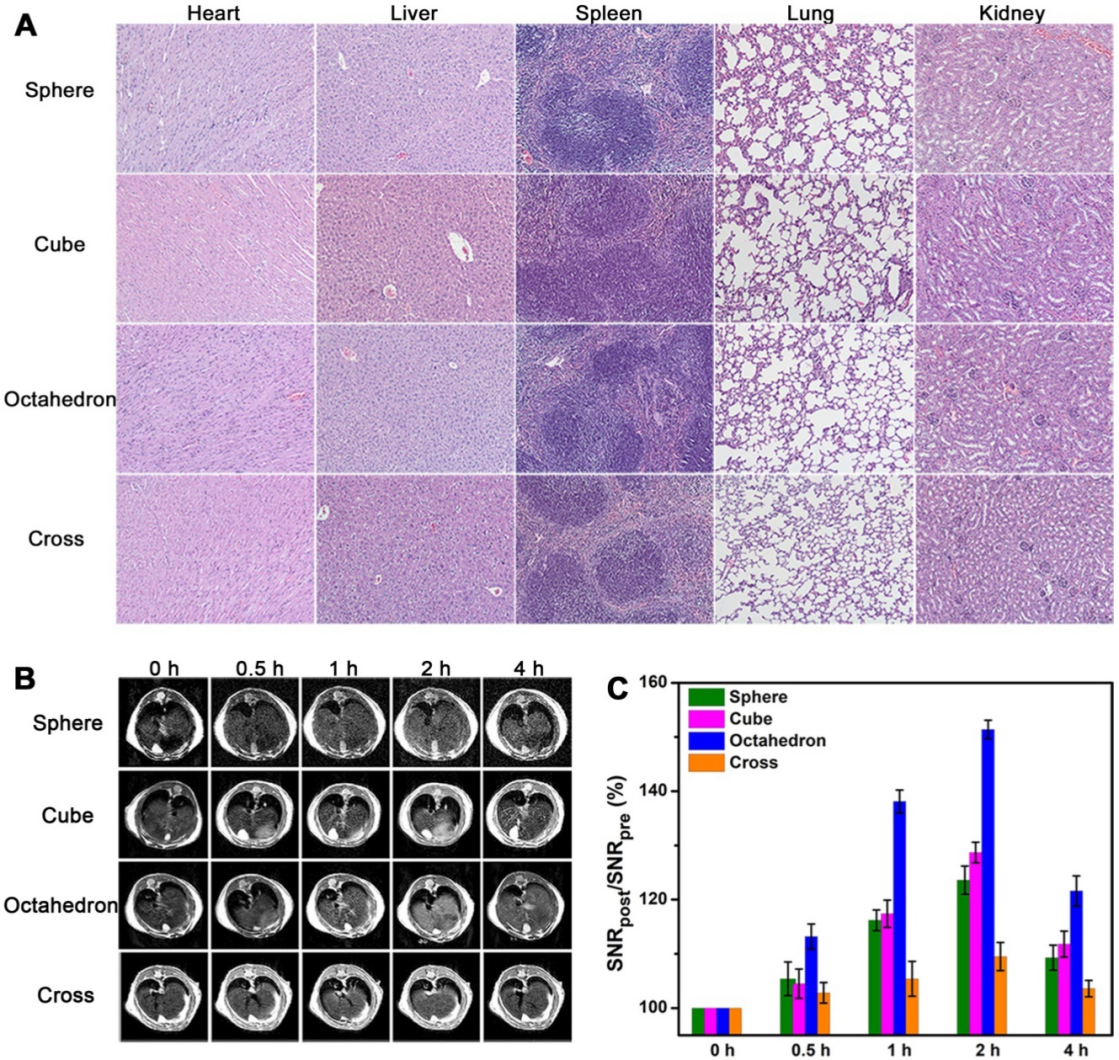
***In vivo******T*_1_ CE-MR imaging of liver. (A)** The organ histology images of different organs (heart, liver, spleen, lung and kidney) after administration of ZDS coated MnO nanoparticles. The H&E staining of BALB/c mice were sacrificed two weeks after caudal venous injection of MnO nanoparticles with four different shapes at a dose of 2.0 mg [Mn]/kg. **(B)** *In vivo T*_1_-weighted MR images in liver at transverse plane of mice before and after intravenous injection of MnO nanoparticles with a dose of 2.0 mg [Mn]/kg to mouse body weight (*n* = 3/group). **(C)** Corresponding quantitative analysis of SNR changes in liver of (B) at different time points after administration (*n* = 3/group).

Before *in vivo T*_1_ imaging, we evaluated the cytotoxicity and biocompatibility of these MnO NPs with different shapes first. The cytotoxicity of these four samples were measured by 3-(4,5-dimethylthiazol-2-y1)-2,5-diphenyltetrazolium bromide (MTT) assays (**Figure S10**). No apparent cytotoxicity was observed in these groups even at the high concentration of 120 μg [Mn]/mL after incubation with human hepatoma SMMC-7721 cells for 24 h. Moreover, H&E staining results (**Figure 6A**) indicated no appreciable tissue injury or inflammation of the major five organs two weeks after venous injection of these MnO NPs for mice (at a dose of 2.0 mg [Mn]/kg). All these results attest to the excellent stability and biocompatibility of ZDS modified MnO NPs.

We established the healthy BALB/c mice model and performed *T*_1_-weighted MRI in liver at 7.0 T by utilizing MnO NPs with four shapes. We focused on the liver as the region of interest, as the majority of NPs are expeditiously taken up by mononuclear phagocyte system (MPS) and rapidly accumulated in hepatic Kupffer cells 53. We obtained* T*_1_-weighted MR images of transverse plane (**Figure 6B**) before and after intravenous injection of NPs at a dose of 2.0 mg [Mn]/kg mouse body weight (*n* = 3/group). Signal enhancements were observed in the liver region for all groups at 0.5, 1, 2, and 4 h post injection. The signals in liver became positive at 0.5 h post injection, reached the brightest at 2 h, and partly recovered at 4 h, implying that NPs were degraded and excreted from the body, which is in agreement with former research 54, 55. In accord with the result of *T*_1_ phantom imaging, spheres and cubes showed clear signal enhancement while cross exhibited little signal change due to its high *r*_2_/*r*_1_ ratio. Notably, octahedrons displayed significantly enhanced signal at 2 h, indicating their towering enhanced ability for *in vivo T*_1_ imaging. Moreover, for octahedrons, the liver region showed a distinct bright signal at 1 h and remained positive until 4 h. They provide hours of diagnostic time window after intravenous administration, which is able to provide more opportunities for gathering critical information for sensitive diagnosis and imaging mediated therapy.

We then analyzed signal-to-noise ratio (SNR) changes to quantify the enhanced effects of these MnO NPs. We calculated the SNR_post_/SNR_pre_ value for the liver region at the transverse plane for each group (**Figure 6C**). Their signal changes (∆SNR%) at 2 h were 23.6 ± 2.6, 28.7 ± 1.9, 51.4 ± 1.7 and 9.5 ± 2.6 for spheres, cubes, octahedrons and cross, respectively (**Table S9**). Consistent with their performance in *in vitro* and* in vivo T*_1_ imaging, the signal changes in liver from small to large were cross, spheres, cubes, and octahedrons. In particular, the maximal ΔSNR at 2 h of octahedrons was up to 51.4%, which is about 2.2 times of spheres, 1.8 times of cubes and 5.4 times of cross. They exhibited the optimal *T*_1_ enhanced effect, further demonstrating that the brilliant contrast ability is due to their high *T*_1_ relaxivity and low *r*_2_/*r*_1_ ratio. Therefore, the accumulation of NPs with this unique octahedron shape in the liver performed an eminent enhanced effect for *T*_1_ MRI.

### *In vivo* behavior

Diverse surface coating ligands could influence NPs' distribution in tissues and *in vivo* fates 56-58. Zwitterionic NPs are able to reduce the non-absorption of proteins in physiological environment 59, maximize tumor accumulation and cellular uptake due to their switchable charges based on the environmental stimulus 60, 61. MnO NPs with zwitterionic ZDS coating exhibit charge switchable behavior, their negatively charged surface may reduce the nonspecific protein adsorption in blood and increase the accumulation in solid tumor sites through the enhanced permeability and retention (EPR) effect 62, and their charge can change to positive by diminishing the anionic part after arriving in tumoral acidic microenvironment, which promotes the tumor cellular uptake and hence increase the diagnosis accuracy 63.

**Figure 7:**
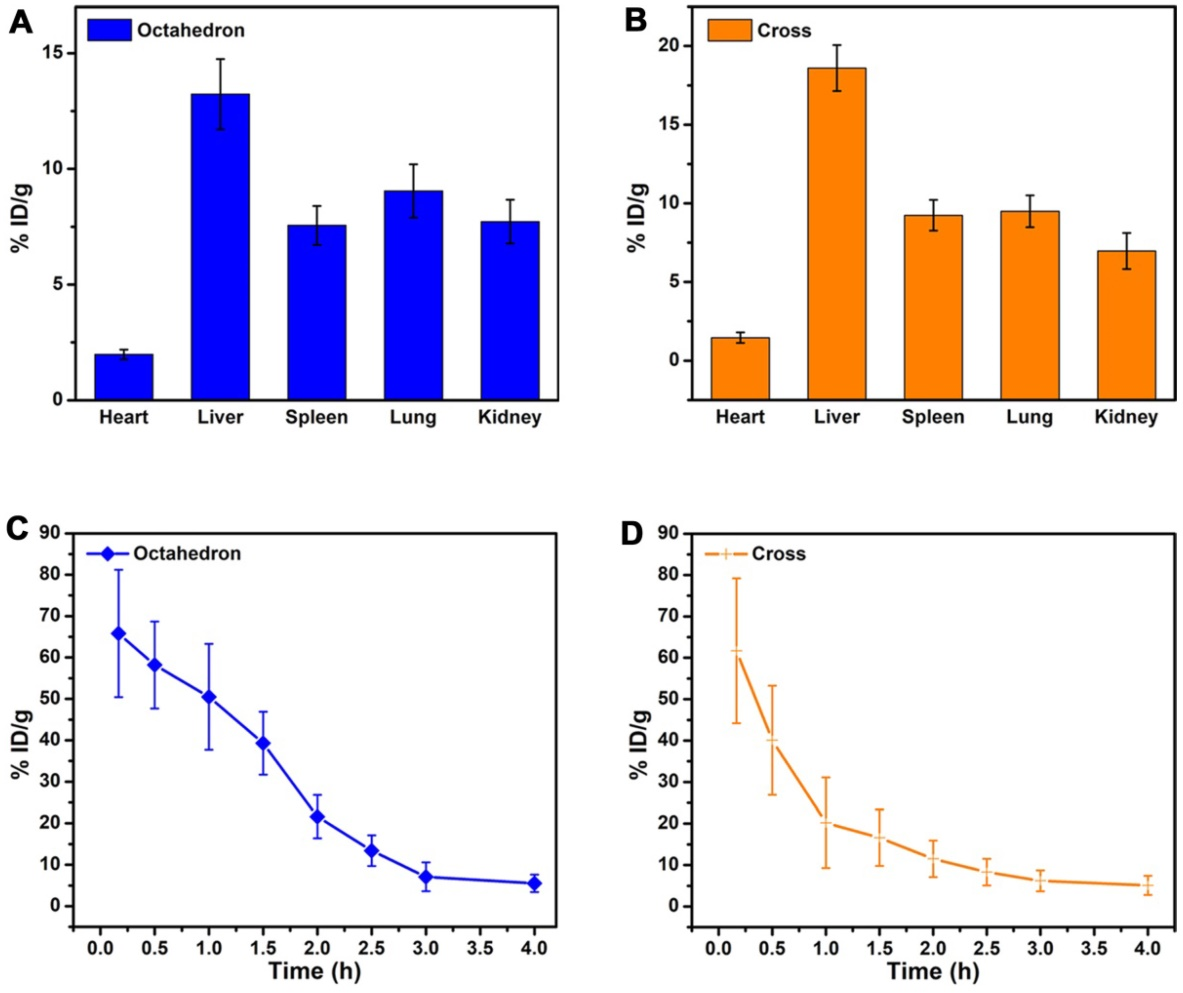
***In vivo* behavior analysis.** *In vivo* biodistribution of Mn ions in major organs of mice at 6 h after intravenous injection of **(A)** MnO octahedrons and **(B)** MnO cross (*n* = 3/group). Blood circulation curves of **(C)** MnO octahedrons and **(D)** MnO cross in mice. The concentrations of Mn ions were measured by ICP-MS (*n* = 3/group).

To investigate pharmacokinetics and diagnosis efficiency, we studied the *in vivo* behavior of MnO NPs by analysis of biodistribution and blood circulation half-life. We noticed that octahedrons performed obvious *T*_1_ enhancement while cross displayed inconspicuous contrast effect. Hence octahedrons and cross were used for comparison to explore the potential reason for this discrepancy. We analyzed *in vivo* biodistribution of octahedrons and cross by detecting the concentration of Mn ions in major organs: heart, liver, spleen, lung and kidney (**Figure 7A-B**). As we previously expected, large part of octahedrons and cross apparently accumulated in the mononuclear phagocyte system such as liver and spleen. The accumulation of cross in liver, spleen and lung was higher while in heart and kidney was lower (**Table S10**), which is owing to the larger hydrodynamic diameters of cross than octahedrons 64. Mononuclear phagocytic cells present in tissues of the liver, spleen, lungs 65, and Kupffer cells that line the hepatic sinusoids in the liver, together with marginal zone and red pulp macrophages in the spleen, rapidly sequester particles with larger hydrodynamic diameters 66. In addition, the accumulation of octahedrons in liver was lower than that of cross, which suggests that the predominant enhanced effect in liver of octahedrons is due to their high *T*_1_ contrast ability.

We further studied the blood circulation half-life of MnO octahedrons and cross (**Figure 7C-D**). The blood circulation half-life of octahedrons is about 1.54 h, which is 2.1 times as long as that of cross with a value of 0.73 h (**Table S11**). This result proves that both octahedrons and cross are stable during circulation because free Mn ions possess a short half-life of only several minutes in blood circulation. It is reported that smaller particles absorb less amounts of proteins in comparison with larger particles of the same material, hence the concentration of particles circulating in the blood decreases with the size increases, since larger particles are prone to be trapped by MPS 67. Octahedrons hold a prolonged *in vivo* circulating half-life, which suggests that octahedrons have reduced nonspecific adsorption of proteins and agglomeration of particles in the blood circulation. This result certifies that MnO octahedrons are more appropriate for detecting tumors compared to MnO cross.

### Sensitive detection of hepatic and subcutaneous tumors

**Figure 8:**
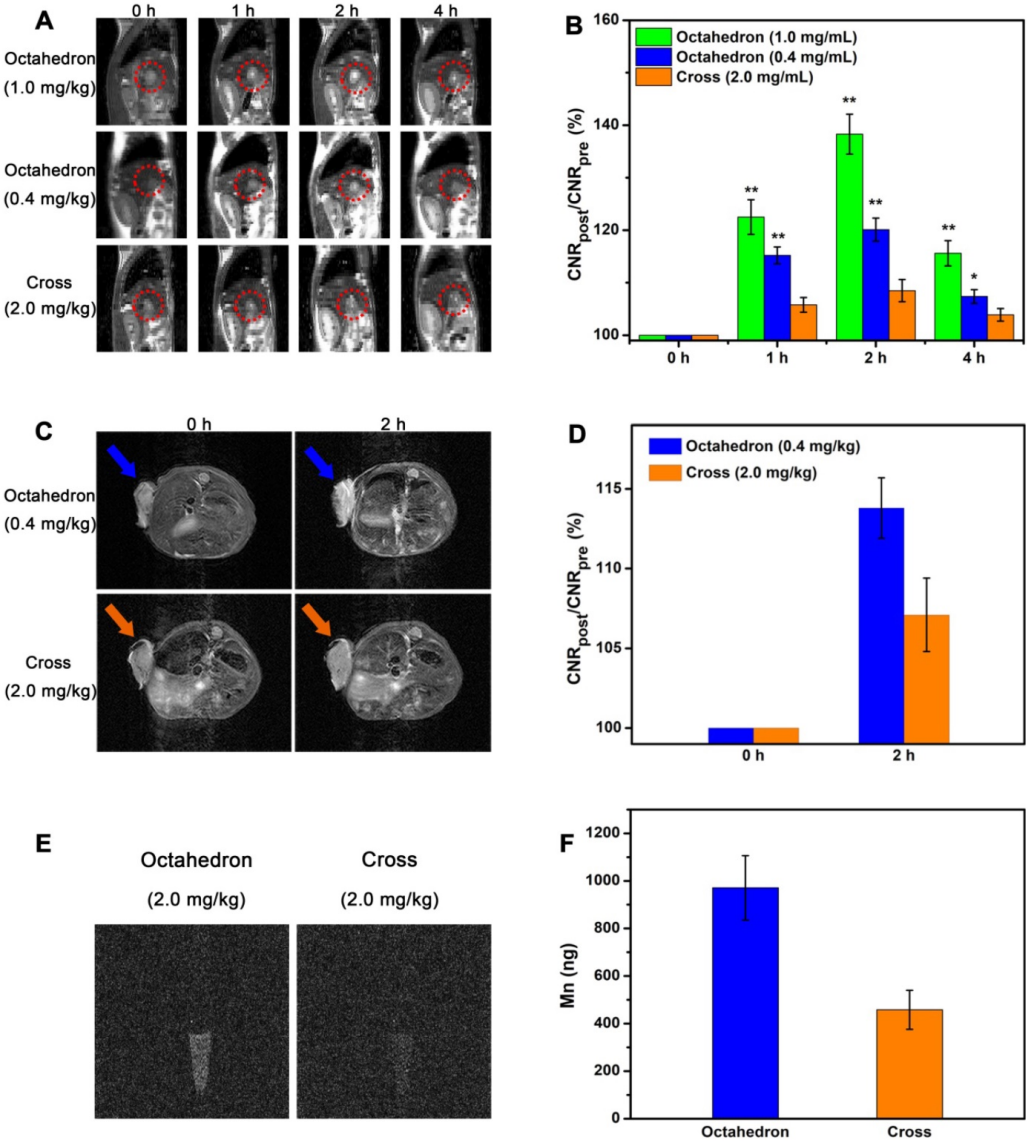
**Sensitive tumor imaging with ultralow dose. (A)** *In vivo T*_1_-weighted MR images of orthotopic liver tumors (in red circles) of BALB/c mice in sagittal plane. **(B)** The corresponding quantitative CNR changes of tumors (*: 0.01 < p < 0.05, **: p < 0.01, *n* = 5/group, compared with the group of cross). **(C)** *T*_1_-weighted MR images of mice bearing subcutaneous tumors at 0 h and 2 h after intravenous injection (*n =* 3/group).** (D)** The related quantification of CNR post administration. **(E)** *T*_1_ imaging of H22 cells isolated from the mice treated with octahedrons and cross after 2 h intravenous injection. **(F)** Total amount analysis of Mn ions in H22 cells isolated from the mice treated with octahedrons and cross.

On the basis of the above *in vivo* MRI results, MnO octahedrons performed superior MR enhancement, suggesting they are potential candidates for sensitive tumor diagnosis. To study their imaging ability for tumors, we conducted *T*_1_ CE-MRI of BALB/c mice bearing orthotopic hepatocellular carcinoma tumors at 7 T. Sagittal images were acquired before (0 h) and at 1, 2, and 4 h after intravenous injection of octahedrons, cross and Mn-DPDP (**Figure 8A** and**Figure S11A**). The injection dose of cross was 2.0 mg [Mn]/kg, the injection doses of octahedrons were 1.0 mg [Mn]/kg and 0.4 mg [Mn]/kg, and the injection dose of Mn-DPDP was 4.0 mg [Mn]/kg. The signals of hepatic tumors for all groups became positive at 1 h, reached the brightest at 2 h, and dimed gradually at 4 h. However, octahedrons showed brighter signals than cross at only a half and one-fifth of the injected dose, and displayed brighter signals than Mn-DPDP at even one-tenth of the injected dose. The remarkably enhanced signals with this ultralow dose affirm the high sensitivity of MnO octahedrons in detecting tumors. Moreover, notably, the dose of 0.4 mg [Mn]/kg for mouse is equal to a human dose of 0.03 mg/kg, which is about 1/10 of the clinical dose of 0.275 mg/kg for Mn-DPDP for human (based on an equivalent surface area dose) (details see **Table S12**) 63, 68, 69. This ultralow dose used indicates less toxicity and side effects in clinical application.

The MR contrast-to-noise ratio (CNR) changes of tumor (**Figure 8B** and**Figure S11B**) further validate their contrast abilities for tumor imaging. The ∆CNR% of octahedrons at a dose of 1.0 mg [Mn]/kg at 1, 2, and 4 h after intravenous injection were 22.5 ± 3.3, 38.3 ± 3.8 and 15.6 ± 2.4, respectively. The ∆CNR% of octahedrons at a dose of 0.4 mg [Mn]/kg at 1, 2, and 4 h after intravenous injection were 15.2 ± 1.6, 20.1 ± 2.2 and 7.4 ± 1.3, respectively. The ∆CNR% of cross at a dose of 2.0 mg [Mn]/kg at 1, 2, and 4 h after intravenous injection were 5.8 ± 1.4, 8.5 ± 2.1 and 3.9 ± 1.2, respectively. The ∆CNR% of Mn-DPDP at a dose of 4.0 mg [Mn]/kg at 1, 2, and 4 h after intravenous injection were 10.3 ± 1.5, 15.2 ± 2.3 and 5.8 ± 1.4, respectively (**Table S13**). The ∆CNR% of octahedrons at a dose of only 0.4 mg [Mn]/kg were evidently higher than that of cross at a dose of 2.0 mg [Mn]/kg and that of Mn-DPDP at a dose of 4.0 mg [Mn]/kg at 2 h after intravenous injection. This result indicates that the significantly enhanced effect is ascribed to their excellent *T*_1_ contrast ability of for liver tumors, suggesting MnO octahedrons is an efficient CA for sensitive diagnosis of hepatic tumor.

We also conducted *T*_1_ CE-MRI of BALB/c mice bearing subcutaneous tumors to investigate their imaging abilities. Considering their excellent *T*_1_ contast ability, we intravenously injected MnO octahedrons only at a dose of 0.4 mg [Mn]/kg to body weight. According to the previous fact that MR signals for mice reach the brightest at 2 h post injection (the best detection window), *T*_1_ CE-MR images were acquired before and at 2 h after intravenous injection (**Figure 8C** and**Figure S12A**). To evaluate their tumor targeting capabilities, we focused on tumors as interesting regions. The tumor showed a significant positive signal at 2 h than that at 0 h, indicating their excellent *T*_1_ contrast ability for subcutaneous tumors. This result confirms that MnO octahedrons with ZDS coating can effectively accumulate in tumor sites, result in obvious differentiation between the tumor and normal tissues in MR images, and thus are suitable in cancer diagnosis. Moreover, in comparison with the group of cross and the group of Mn-DPDP, octahedrons showed a brighter contrast enhanced signal at 2 h than that of cross with only 1/5 of the injected dose and that of Mn-DPDP with 1/10 of the injected dose.

To quantify their contrast enhancements, we measured SNR values of the three groups by analyzing the region of images corresponding to tumors (**Figure S13**). Compared with the SNR value of 107.5 ± 2.1% for the cross group and the SNR value of 109.1 ± 2.2% for the Mn-DPDP group, the octahedron group shows a higher SNR value of 112.9 ± 2.4% at 2 h. The high *T*_1_ signal enhancement in the octahedron group indicates that octahedrons is an efficient candidate for sensitive tumor diagnosis. Another key factor to evaluate the enhanced effect, the MR contrast-to-noise ratio (CNR) change of tumor (**Figure 8D** and **Figure S12B**), further confirms their contrast abilities for tumor imaging. Consistent with SNR analysis, the ∆CNR% of octahedrons at a dose of 0.4 mg [Mn]/kg was 13.8 ± 1.9, which is much higher than that of cross with a value of 7.1 ± 2.3 at a dose of 2.0 mg [Mn]/kg and that of Mn-DPDP with a value of 10.4 ± 2.3 at a dose of 4.0 mg [Mn]/kg.

### *In vivo* tumor uptake

Since the *T*_1_ signal enhancement of tumor is proportional to the number of accumulated particles in tumor sites, we studied the tumor uptake of MnO octahedrons and MnO cross (**Figure S14**). The tumor uptake of octahedrons and cross was 2.19 ± 0.75% ID/g and 4.76 ± 1.08 % ID/g. It is noted that the injection dose of octahedron was merely 1/5 of that of cross, while the tumor uptake of octahedron was nearly half of that of cross, which indicates the high tumor uptake of octahedrons.

We also investigated the *in vivo* cellular uptake of tumor on mice bearing H22 tumors by the same injection dose of 2.0 mg [Mn]/kg octahedrons and cross. The cells were isolated from the mice and then conducted by *T*_1_-weighted MR imaging. The group treated by octahedrons exhibited a clear *T*_1_ signal, while the signal in the group treated by cross was obscure (**Figure 8E**). Agrees with the results of MR imaging, the amount of Mn ions in cells of the group treated by octahedrons with a value of 971 ± 135 ng is two times higher than that of the group treated by cross with a value of 458 ± 82 ng through ICP-MS analysis (**Figure 8F**). This result proves that MnO octahedrons can effectively accumulate in solid tumors and be taken by tumor cells, which is because that particle size also influences the cellular uptake of NPs via various pathways such as receptor-mediated or adsorptive endocytosis 70-72. The above tumor uptake and signal change verify the excellent *T*_1_ enhanced effect of MnO octahedrons for tumors even at 1/10 of the clinical dose, which can be applied to achieve lower detection limit and less side effects with low dose. These results confirm that MnO octahedrons can improve the sensitivity of *T*_1_ imaging and this feature is crucially important for sensitive detection and early diagnosis of cancer.

## Discussion and Conclusions

SBM theory reveals the relationships of rotation correlation time (τ_R_), hydration number (q), proton residency time (τ_M_) and relaxation time of electron spins (τ_s_) with the *T*_1_ relaxivity for complex, but only the coordinating water molecular number q is applicative for the analysis of NPs. Besides, most of previous reports investigate one or two aspects that influence the enhanced effect of *T*_1_ imaging for nanomaterials. Hence, our work replenishes SBM theory and is complementary to former researches. It takes into account NPs of different shapes from 0D to 3D and also be feasible to other sophisticate shapes.

In summary, we studied critical factors that improved the sensitivity of *T*_1_ imaging by utilizing MnO NPs of different sizes and shapes with neutral charges as research subjects. We demonstrated that geometric volume, surface area, crystal face and *r*_2_/*r*_1_ ratio had a pivotal impact on the enhanced effect of *T*_1_ imaging. The sensitive *T*_1_ imaging has a positive relationship with surface area and occupancy rate of effect metal ions, while a negative correlation with geometric volume and *r*_2_/*r*_1_ ratio. This work figures out quantitative relationships of these factors that influence the enhanced effect of *T*_1_ imaging for the first time. With a systematical understanding, we believe this study would greatly contribute to more rational design and development of high-performance CA for early and precise diagnosis.

Compared with other reported MnO NPs, MnO octahedrons have a notable high *r*_1_ value with a low *r*_2_/*r*_1_ ratio and exhibit remarkable *T*_1_ enhanced effect, holding great promise as an outstanding *T*_1_ CA for sensitive imaging and diagnosis. Their extraordinary *T*_1_ enhanced ability is due to their specific crystal surface and surface area to volume ratio, which are highly related to the shape. Meanwhile, MnO cross maybe a promising candidate for *T*_2_ imaging owing to its large *r*_2_/*r*_1_ ratio. On this basis of these results, it is expected to realize better utilization of nanomaterials with optimal shape and make more breakthroughs in this field.

Moreover, the prominent contrast ability of MnO octahedrons endows an excellent enhanced effect in *in vitro* and *in vivo* liver *T*_1_ MRI. Zwitterionic ZDS coating with charge switchable behavior promotes tumor accumulation and cellular uptake, hepatic and subcutaneous tumors can be detected with an ultralow dose of MnO octahedrons, which is critical for early diagnosis and sensitive prognosis in cancer management. Furthermore, these MnO octahedrons could meet the diagnostic needs even at superb low doses, which is of great importance for reducing the medical costs and side effects in clinic.

## Supplementary Material

Supplementary figures and tables.

## Abbreviations

CAs: contrast agents
CE: contrast-enhanced
CNR: contrast-to-noise ratio
DLS: dynamic light scattering
EDX: energy-dispersive X-ray
EPR: enhanced permeability and retention
FDA: Food and Drug Administration
HRTEM: high-resolution transmission electron microscopy
ICP-MS: inductively coupled plasma mass spectroscopy
MnO: manganese oxide
MPS: mononuclear phagocyte system
MRI: magnetic resonance imaging
MSE: multislice spin-echo
NPs: nanoparticles
NSF: nephrogenic systemic fibrosis
PDI: polydispersity coefficient
SBM: Solomon, Bloembergen, and Morgan
SNR: signal-to-noise ratio
SQUID: superconducting quantum interference device
TEM: transmission electron microscopy
XPS: X-ray absorption spectra
XRD: X-ray powder diffraction
ZDS: zwitterionic dopamine sulfonate
