# Supplementary material for: Improving the sensitivity of T1 contrast-enhanced MRI and sensitive diagnosing tumors with ultralow doses of MnO octahedrons
Source: Theranostics. 2021 May 8;11(14):6966–82. doi: 10.7150/thno.59096 (PMC8171088; doi:10.7150/thno.59096)
Supplement: Supplementary file 1 — Supplementary figures and tables. [file thnov11p6966s1.pdf]

Supporting Material

Improving the sensitivity of T_1 contrast-enhanced MRI and sensitive diagnosing tumors with ultralow doses of MnO octahedrons

Lijiao Yang,[1#] Lili Wang,[2#] Guoming Huang,[1] Xuan Zhang,[1] Lanlan Chen[1✉] Ao Li,[3] Jinhao Gao[3✉] , Zijian Zhou,[4] Lichao Su,[1] Huanghao Yang,[1] Jibin Song[1✉]

1. MOE Key Laboratory for Analytical Science of Food Safety and Biology, College of Chemistry, Fuzhou University, Fuzhou 350108, China

2. Department of Diagnostic Radiology, Fujian Medical University Union Hospital, Fuzhou 350001, P. R. China

3. State Key Laboratory of Physical Chemistry of Solid Surfaces, The MOE Laboratory of Spectrochemical Analysis & Instrumentation, and Department of Chemical Biology, College of Chemistry and Chemical Engineering, Xiamen University, Xiamen 361005, China

4. State Key Laboratory of Molecular Vaccinology and Molecular Diagnosis & Center for Molecular Imaging and Translational Medicine, School of Public Health, Xiamen University, Xiamen 361102, China

#These authors contributed equally to this work.

✉Corresponding authors: llchen@fzu.edu.cn; jhgao@xmu.edu.cn; jibinsong@fzu.edu.cn.

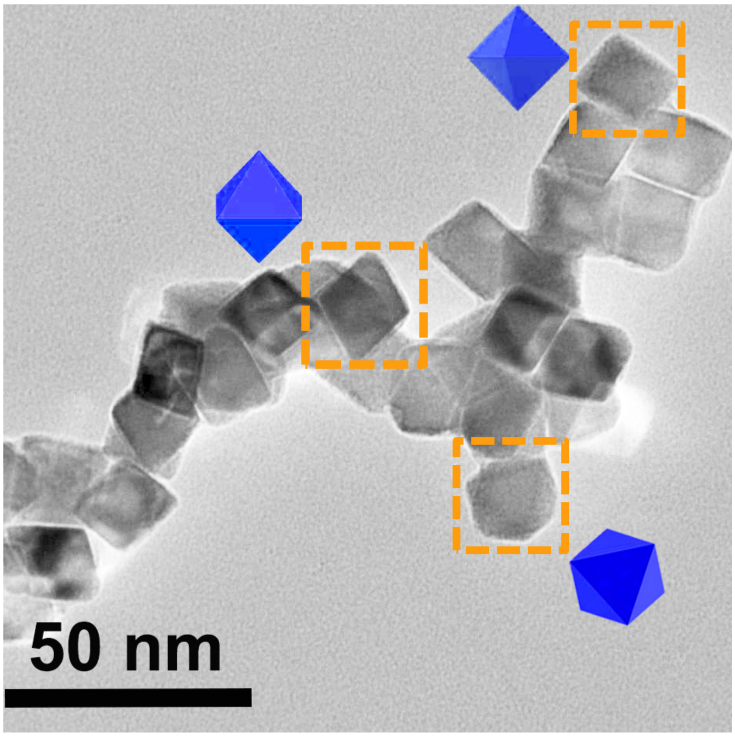

Figure S1. The confirmation of the octahedra in TEM image. Each picture with certain orientation showed the shape of the nanoparticle inside the box.

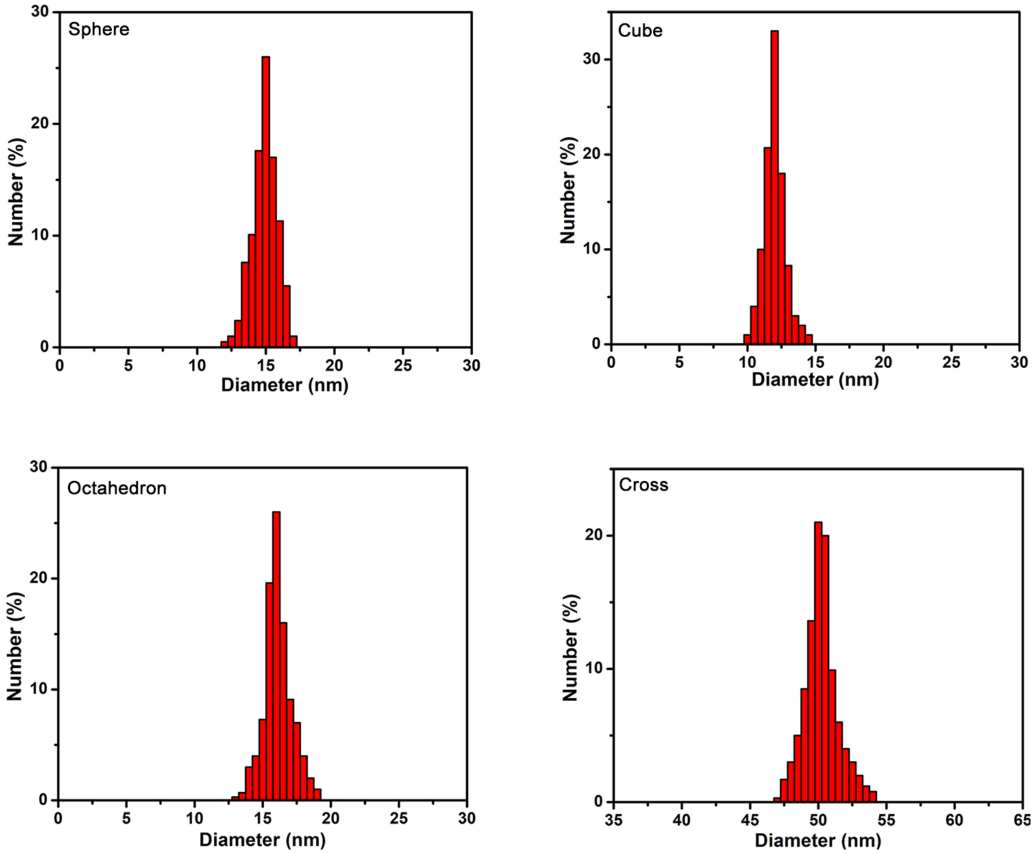

Figure S2. The diameter histograms of as-synthesized MnO nanoparticles with four different shapes. The size distributions were acquired by measuring at least two hundred particles per sample *via* Image J.

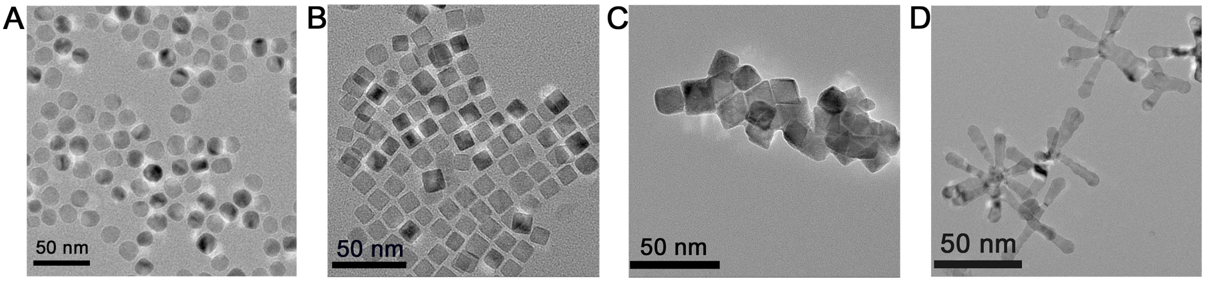

Figure S3. TEM images of ZDS-coated MnO NPs with different shapes. (A) spheres, (B) cubes, (C) octahedrons and (D) cross.

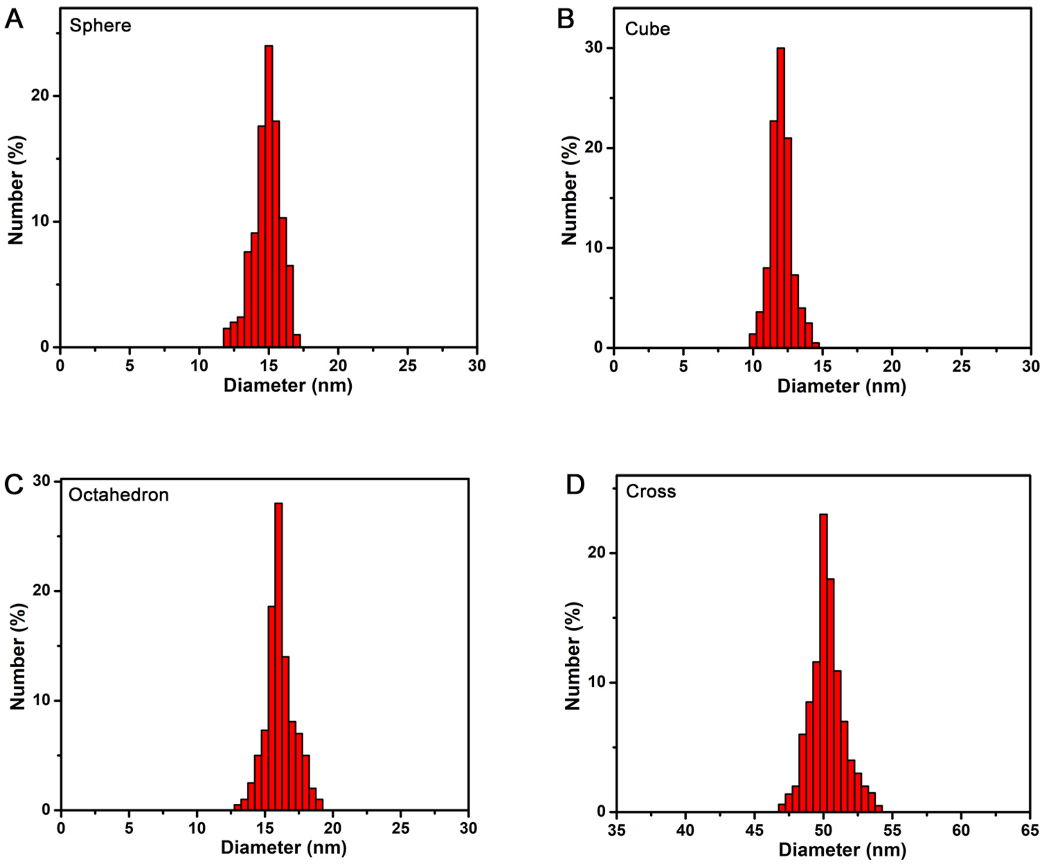

Figure S4. Size distributions of ZDS-coated MnO NPs with different shapes. (A) spheres, (B) cubes, (C) octahedrons and (D) cross. The sizes were obtained by measuring two hundred particles per sample according to Image J analysis.

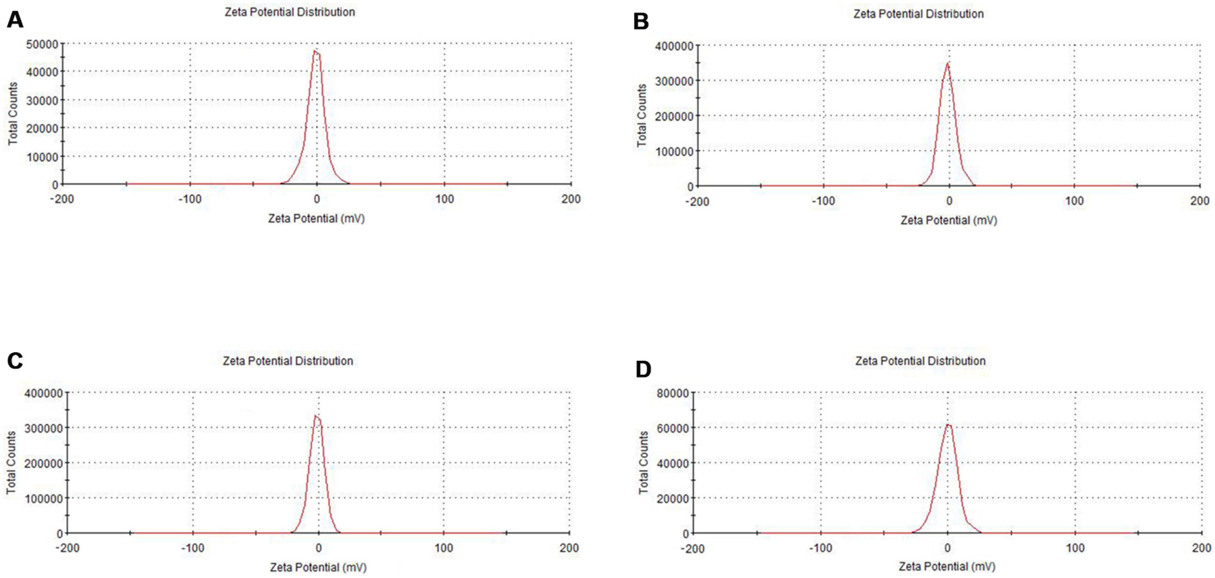

Figure S5. Analyses of the Zeta potential of MnO nanoparticles with diverse shapes. Zeta potentials of **(A)** MnO spheres, **(B)** MnO cubes, **(C)** MnO octahedra and **(D)** MnO cross.

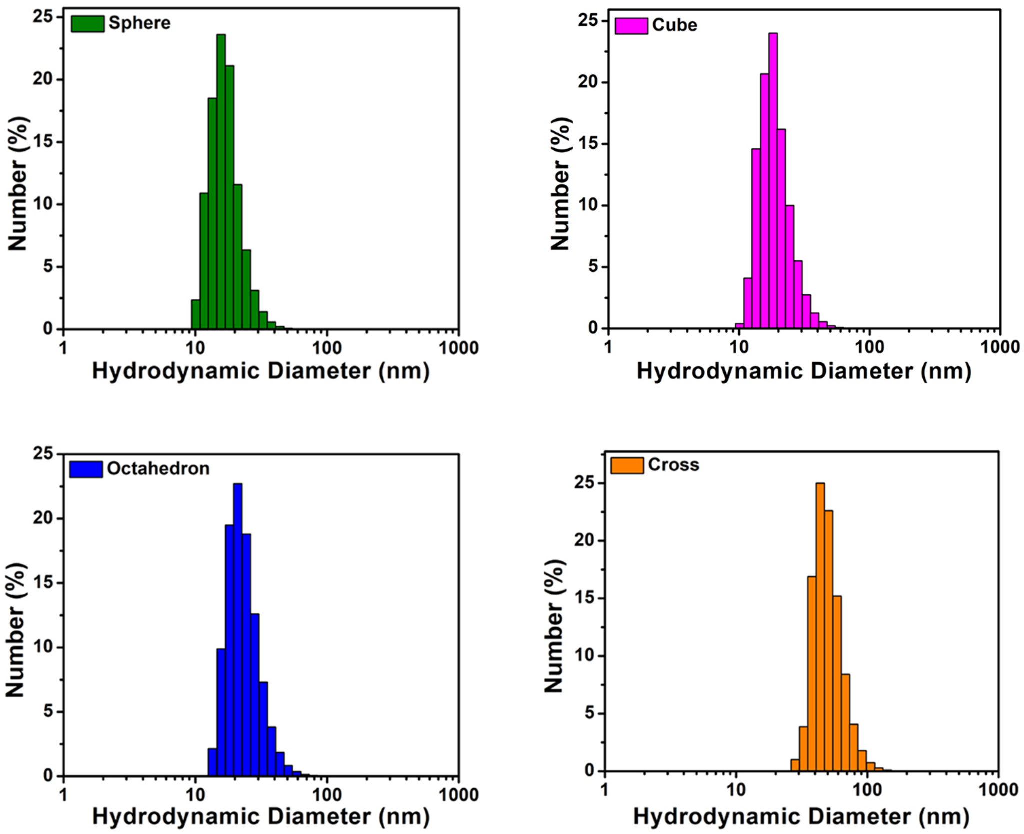

Figure S6. The dynamic light scattering (DLS) analysis. The DLS measurements of water-dispersible MnO nanoparticles after ZDS coating.

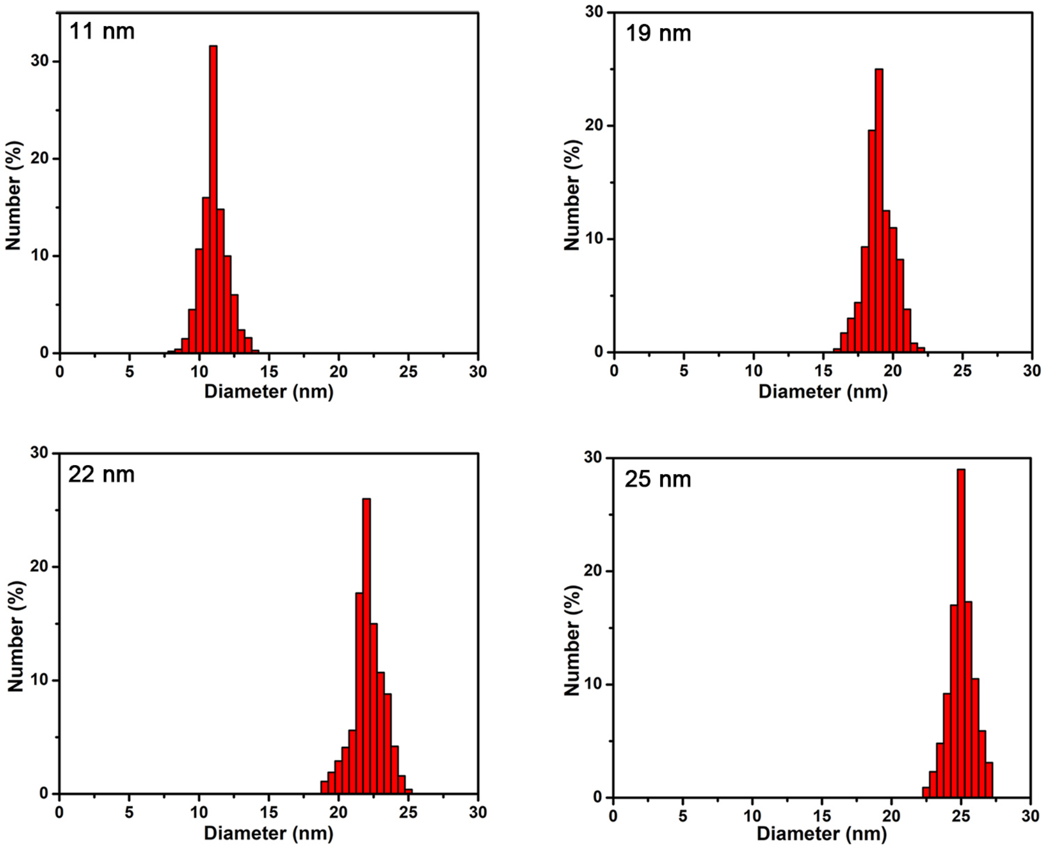

Figure S7. Size distributions. The size histograms of as-synthesized MnO spheres with four different sizes: 11 nm, 19 nm, 22 nm and 25 nm. The diameter distributions were obtained by measuring of two hundred particles per sample.

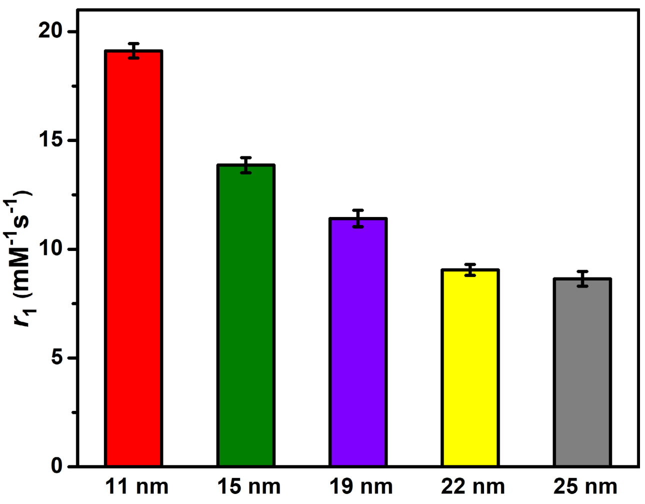

Figure S8. ***T_1* relaxivities of different sizes.** The r_1 values of these MnO spheres with five different sizes: 11 nm, 15 nm, 19 nm, 22 nm and 25 nm.

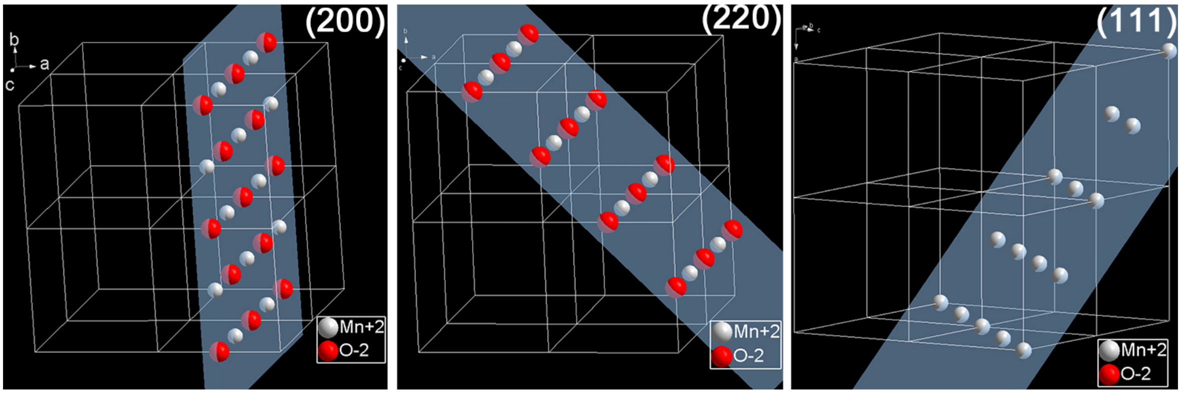

Figure S9. The occupation of metal atoms on exposed surfaces. The occupation rates of manganese and oxygen ions on (200), (220), and (111) surfaces. All the pictures are cut out from the 2*2*2 cells of the MnO crystal structure.

Note about the crystal structure:

Origin: ICSD- 29326.

Name: Manganese oxide.

Formula: Mn O

Space-group: Fm -3m (225) - cubic.

Cell: a = 4.4240 Å, V = 86.59 Å^3 Z = 4

Atomic parameters

Atom	Ox.	Wyck.	Site	S.O.F.	x/a	y/b	z/c	U [Å^2]
Mn1	2	4a	m-3m		0	0	0	
O1	-2	4b	m-3m		1/2	1/2	1/2	

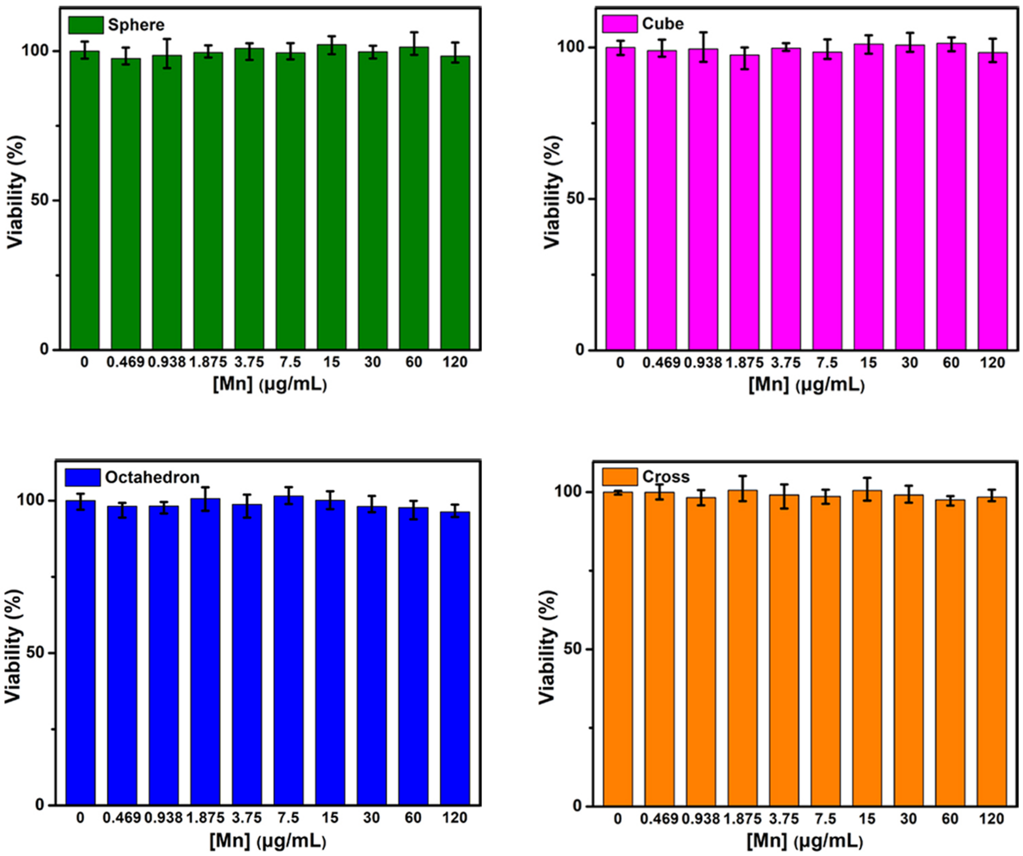

Figure S10. Cytotoxicity evaluations. MTT assay of SMMC-7721 cells incubated with MnO nanoparticles with diverse shapes for 24 h (*n* = 5/group).

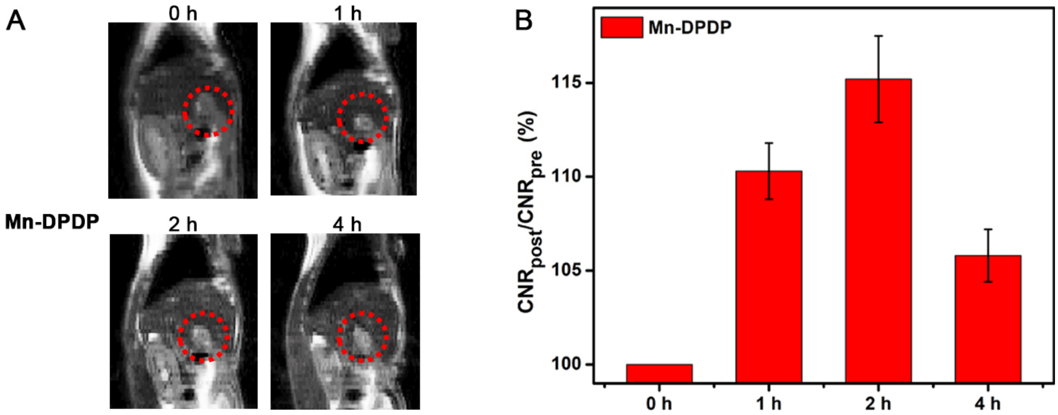

Figure S11. *In vivo* tumor imaging in liver with Mn-DPDP. (A) T_1 CE-MR images of orthotopic liver tumors (in red circles) of BALB/c mice in the sagittal plane with Mn-DPDP at a dose of 4.0 mg [Mn]/kg. **(B)** The related quantitative CNR changes of tumors.

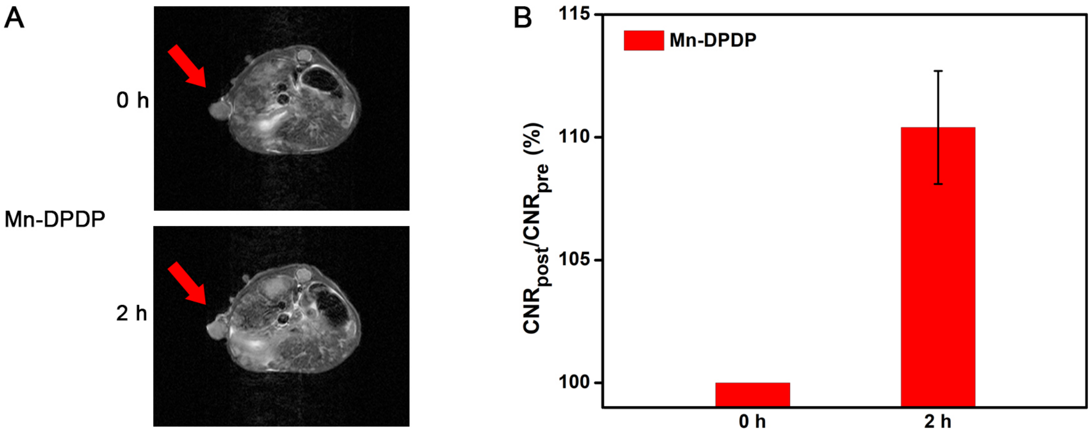

Figure S12. Subcutaneous tumor imaging with Mn-DPDP. (A) T_1-weighted MR images of mice bearing subcutaneous tumors at 0 h and 2 h after intravenous injection with Mn-DPDP at the dosage of 4.0 mg [Mn]/kg (n = 3/group). **(B)** The corresponding quantification of CNR post administration.

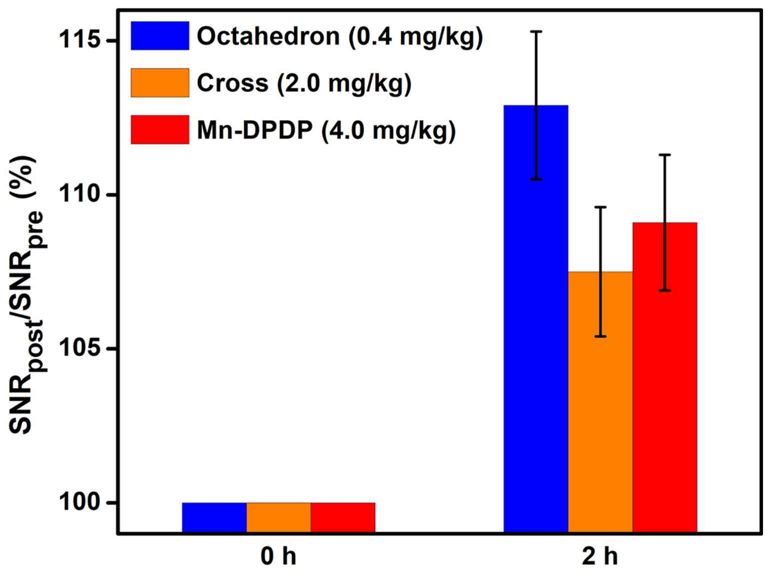

Figure S13. Signal change analysis. The related quantification analysis of signal change of tumor (SNR_{tumor}) in Figure 8A and Figure S11A at 0 h and 2 h after intravenous injection of MnO octahedrons (0.4 mg [Mn]/kg), MnO cross (2.0 mg [Mn]/kg) and Mn-DPDP (4.0 mg [Mn]/kg) (*n* = 3/group).

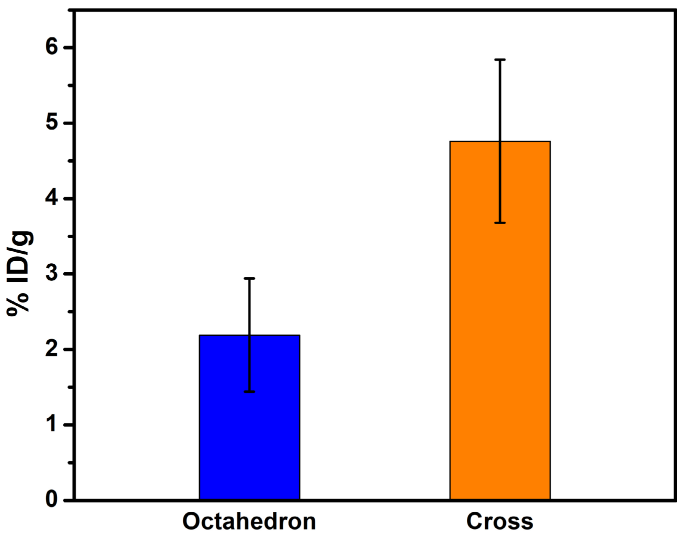

Figure S14. Tumor uptake analysis. The tumor uptake of Mn ions in mice at 2 h after intravenous injection of MnO octahedrons (0.4 mg [Mn]/kg) and MnO cross (2.0 mg [Mn]/kg).

Table S1. Synthetic conditions. The synthetic conditions for MnO nanoparticles with different sizes and shapes.

shape	sphere (11 nm)	sphere (15 nm)	sphere (19 nm)	sphere (22 nm)	sphere (25 nm)	cube	octahedron	cross
Manganese (II) oleate/g	0.618	0.618	0.618	0.618	0.618	0.618	0.618	0.618
sodium oleate/mg	0	0	0	0	0	0	0.061	0.152
oleic acid /mL	0.161	0.161	0.161	0.161	0.161	0.161	0.161	0.322
1-octadecene /mL	10	10	10	10	10	15	12	15
atmosphere	N_2	N_2	N_2	N_2	N_2	N_2	N_2	N_2
heating process	100 °C (20 min), 200-250 °C (30 min), slowly to 320 °C	100 °C (20 min), 200-250 °C (30 min), slowly to 320 °C	100 °C (20 min), 200-250 °C (30 min), slowly to 320 °C	100 °C (20 min), 200-250 °C (30 min), slowly to 320 °C	100 °C (20 min), 200-250 °C (30 min), slowly to 320 °C	100 °C (20 min), rapidly to 330 °C	100 °C (20 min), rapidly to 350 °C	to 300 °C (5 °C min^{-1})
reflux time	1 h	1.5 h	2 h	2.5 h	3 h	2 h	1.5 h	4 h

Table S2. Stability of water-soluble MnO nanoparticles. The hydrodynamic diameter and the polydispersity coefficient (PDI) of ZDS coated MnO nanoparticles with different shapes analyzed by dynamic light scattering (DLS). Measurements were carried out after MnO nanoparticles being stored for more than six months.

Shape	Hydrodynamic Diameter (nm)	PDI
Sphere	16.25 ± 1.92	0.193
Cube	19.98 ± 3.01	0.248
Octahedron	23.17 ± 2.38	0.215
Cross	51.34 ± 3.94	0.259

DLS: dynamic light scattering; PDI: polydispersity coefficient.

Table S3. Relationships of diameters, surface to volume ratios and T_1 relaxivities. The relationships of diameters, surface to volume ratios and T_1 relaxivities and of MnO spheres with various diameters at 0.5 T.

Diameter (nm)	r_1 ($mM^{-1}s^{-1}$)	Surface to Volume (nm^{-1})
11	19.12 ± 0.33	0.54
15	13.86 ± 0.35	0.40
19	11.41 ± 0.38	0.32
22	9.05 ± 0.25	0.27
25	8.64 ± 0.34	0.24

Note：Details about the Linear Fit and Nonlinear Curve Fit.

1. The function of r_1 values and diameters of MnO spheres with different sizes.

Model: Allometric1

Equation: y = a*x^b

Adj. R-Square 0.99183

	Value	Standard Error
a	210.13295	25.7586
b	-1.00031	0.04526

2. The function of r_1 values and surface to volume ratios of MnO spheres.

Equation: y = a + b*x

Pearson's r 0.99791

Adj. R-Square 0.99444

	Value	Standard Error
a	-0.17924	0.49169
b	35.57977	1.32889

Table S4. The occupancy rates of ions. The occupancy rates of manganese and oxygen ions on different exposed faces (a is the side length of the unit cell).

face	atom	(200)	(220)	(111)
occupancy rates	Mn	2	1.41	2.31
(per a^2)	O	2	1.41	0

Note: The different occupancy rates of manganese and oxygen ions on different faces (in 2×2×2 cells, see Figure S7)

For (111), $S = \frac{\sqrt{3}}{4} \times (2\sqrt{2}a)^2 = 2\sqrt{3}a^2$

$n(Mn^{2+}) = 3 + 9\times\frac{1}{2} + 3\times\frac{1}{6} = 8$, $Mn^{2+} = n/S = 2.31$ (per a^2)

For (200), $S = (2a)^2 = 4a^2$

$n(Mn^{2+}) = 4 + 8\times\frac{1}{2} = 8$, $Mn^{2+} = n/S = 2$ (per a^2)

$n(O^{2-}) = 5 + 4\times\frac{1}{2} + 4\times\frac{1}{4} = 8$, $O^{2-} = n/S = 2$ (per a^2)

For (220), $S = 2a \times \frac{3\sqrt{2}}{2}a = 3\sqrt{2}a^2$

$n(Mn^{2+}) = 4 + 4\times\frac{1}{2} = 6$, $Mn^{2+} = n/S = 1.41$ (per a^2)

$n(O^{2-}) = 2 + 6\times\frac{1}{2} + 4\times\frac{1}{4} = 6$, $O^{2-} = n/S = 1.41$ (per a^2)

Table S5. **The geometrical volumes of MnO with different shapes.**

Shape	Sphere	Cube	Octahedron	Cross
Volume (nm^3)	1767	1728	1931	1865

Note: The length or diameter for each shape was an average calculated from two hundred nanoparticles for every sample.

Define the side length as **a** (nm) or the length as **l** (nm), the diameter as **d** (nm) of all the MnO nanoparticles. The theoretical geometrical volume of various shapes are as follows:

sphere: d = 15 nm, $V = \frac{\pi}{6}d^3 = 1767\ nm^3$

cube: a = 12 nm, $V = a^3 = 1728\ nm^3$

octahedron: a = 16 nm, $V = \frac{\sqrt{2}}{3}a^3 = 1931\ nm^3$

cross: d = 5 nm, l (length) = 50 nm, $V = \frac{\pi}{4}d^2(2l - d) = 1865\ nm^3$

Table S6. **The surface area of MnO with different shapes.**

Shape	Sphere	Cube	Octahedron	Cross
Surface area (nm^2)	707	864	887	1492

Note: Define the side length as a (nm) or the diameter as d (nm) of all the nanoparticles

sphere: $d = 15$ nm, $S = \pi d^2 = 707$ nm^2

cube: $a = 12$ nm, $S = 6a^2 = 864$ nm^2

octahedron: $a = 16$ nm, $S = 2\sqrt{3}a^2 = 887$ nm^2

cross: $d = 5$ nm, l (length) = 50 nm, $S = \pi d^2 + 2\pi d\,(l - d) = 1492$ nm^2

Table S7. The relationships of occupancy rates of manganese (n), surface area (S), volume (V) and T_1 relaxivities of MnO nanoparticles with different shapes.

shape	volume (V)	surface area (S)	occupancy rate (n)	nS/V	r_1
sphere	1767	707	2.00	0.800	13.86
cube	1728	864	1.41	0.705	12.44
octahedron	1931	887	2.31	1.06	20.07
cross	1865	1492	2.00	1.60	28.99

Note：Details about the Linear Fit of T_1 relaxivities and nS/V of MnO nanoparticles with different shapes.

Equation: y = a + b*x

Pearson's r   0.99678

Adj. R-Square   0.99036

	Value	Standard Error
a	-0.63221	1.16763
b	18.7008	1.06361

Table S8. The T_1 relaxivities, T_2 relaxivities and r_2/r_1 ratios of MnO with different shapes at 0.5 T.

shapes	r_1 ($mM^{-1}s^{-1}$)	r_2 ($mM^{-1}s^{-1}$)	r_2/r_1
sphere	13.86 ± 0.41	38.81 ± 1.41	2.80
cube	12.44 ± 0.38	32.28 ± 1.55	2.59
octahedron	20.07 ± 0.55	39.02 ± 1.28	1.94
cross	28.99 ± 0.64	147.6 ± 1.97	5.09

Table S9. MR signal-to-noise ratio (SNR) changes of liver. SNR changes of liver at transverse plane in T_1 imaging before and after intravenous injection of MnO nanoparticles with four shapes at 7.0 T (n = 3/group). The SNR was calculated by the equation: $SNR_{liver} = SI_{liver}/SD_{noise}$.

	Sphere	Cube	Octahedron	Cross
SNR_{pre} (%)	100	100	100	100
$SNR_{0.5\ h}$ (%)	105.4 ± 3.1	104.5 ± 2.7	113.2 ± 2.3	102.8 ± 1.9
$\Delta SNR_{0.5\ h}$ (%)	5.4 ± 3.1	4.5 ± 2.7	13.2 ± 2.3	2.8 ± 1.9
$SNR_{1\ h}$ (%)	116.2 ± 1.9	117.4 ± 2.5	138.1 ± 2.1	105.4 ± 3.2
$\Delta SNR_{1\ h}$ (%)	16.2 ± 1.9	17.4 ± 2.5	38.1 ± 2.1	5.4 ±3.2
$SNR_{2\ h}$ (%)	123.6 ± 2.6	128.7 ± 1.9	151.4 ± 1.7	109.5 ± 2.6
$\Delta SNR_{2\ h}$ (%)	23.6 ± 2.6	28.7 ± 1.9	51.4 ± 1.7	9.5 ± 2.6
$SNR_{4\ h}$ (%)	109.3 ± 2.3	111.8 ± 2.4	121.6 ± 2.8	103.6 ± 1.5
$\Delta SNR_{4\ h}$ (%)	9.3 ± 2.3	11.8 ± 2.4	21.6 ± 2.8	3.6 ± 1.5

SNR: signal-to-noise ratio

Table S10. *In vivo* biodistribution. Biodistribution of major organs in mice treated with MnO octahedrons and MnO cross (%ID /g).

	Heart	Liver	Spleen	Lung	Kidney
Octahedron	1.98 ± 0.21	13.23 ± 1.52	7.56 ± 0.84	9.05 ± 1.15	7.72 ± 0.95
Cross	1.46 ± 0.33	18.60 ± 1.46	9.24 ± 0.98	9.49 ± 1.01	6.97 ± 1.15

Table S11. Blood circulation half-life analysis. Blood circulation half-life analysis of MnO octahedrons and cross in mice after intravenous injection. The concentrations of Mn ions were measured by ICP-MS (*n* = 3/group).

time	Octahedron (%)	cross (%)
10 min	65.8 ± 15.4	61.7 ± 17.5
0.5 h	58.2 ± 10.5	40.1 ± 13.2
1 h	50.5 ± 12.8	20.2 ± 10.9
1.5 h	39.3 ± 7.6	16.6 ± 6.8
2 h	21.6 ± 5.2	11.5 ± 4.4
2.5 h	13.4 ± 3.7	8.3 ± 3.2
3 h	7.1 ± 3.5	6.2 ± 2.5
4 h	5.5 ± 2.1	5.1 ± 2.3

Table S12. Conversion factors of common species. This table gives approximate factors for converting doses expressed in terms of mg/kg from one species to an equivalent surface area dose expressed as mg/kg in the other species.

species	body weight (kg)	surface area (m^2)	conversion factor (kg/m^2)
mouse	0.02	0.0066	3
rat	0.15	0.025	6
dog	3	0.24	12.5
monkey	8	0.4	20
human (child)	20	0.8	25
human (adult)	60	1.6	37.5

Example: to express a mg/kg dose in any given species as the equivalent mg/m^2 dose, multiply the dose by the appropriate kg/m^2,

in human adults, 0.03 mg/kg is equivalent to 0.4 mg/kg*3 kg/m^2 =1.2 mg/m^2.

To convert a dose in the mouse to an equivalent dose in the human by using the equivalent surface area dosage conversion factor in the table:

the conversion factor of mouse is 3 kg/m^2, the conversion factor of human is 37.5 kg/m^2, assuming equivalency on the basis of mg/m^2, to convert a dose of 0.4 mg/kg in the mouse to an equivalent dose in the human,

the equivalent dose is 0.4 mg/kg*3 kg/m^2/37.5 kg/m^2 = 0.03 mg/kg.

Therefore, the dose of 0.4 mg [Mn]/kg for mouse is equal to a human dose of 0.03 mg [Mn]/kg.

Table S13. Contrast-to-noise ratio (CNR) changes. The corresponding quantitative CNR changes of hepatic tumors at 0 h, 1 h, 2 h and 4 h after intravenous injection of MnO octahedrons, MnO cross, and Mn-DPDP at 7.0 T (n = 3/group). The CNR was calculated by the equation: $CNR = (SNR_{tumor} - SNR_{liver})/SNR_{tumor}$.

	Octahedron (1.0 mg/mL)	Octahedron (0.4 mg/mL)	Cross (2.0 mg/mL)	Mn-DPDP (4.0 mg/mL)
$CNR_{0\ h}$ (%)	100	100	100	100
$CNR_{1\ h}$ (%)	122.5 ± 3.3	115.2 ± 1.6	105.8 ± 1.4	110.3 ± 1.5
$\Delta CNR_{1\ h}$ (%)	22.5 ± 3.3	15.2 ± 1.6	5.8 ± 1.4	10.3 ± 1.5
$CNR_{2\ h}$ (%)	138.3 ± 3.8	120.1 ± 2.2	108.5 ± 2.1	115.2 ± 2.3
$\Delta CNR_{2\ h}$ (%)	38.3 ± 3.8	20.1 ± 2.2	8.5 ± 2.1	15.2 ± 2.3
$CNR_{4\ h}$ (%)	115.6 ± 2.4	107.4 ± 1.3	103.9 ± 1.2	105.8 ± 1.4
$\Delta CNR_{4\ h}$ (%)	15.6 ± 2.4	7.4 ± 1.3	3.9 ± 1.2	5.8 ± 1.4

CNR: quantitative contrast-to-noise ratio
